# Simulation System of Electric-Powered Wheelchairs for Training Purposes

**DOI:** 10.3390/s20123565

**Published:** 2020-06-24

**Authors:** Kevin A. Hernandez-Ossa, Eduardo H. Montenegro-Couto, Berthil Longo, Alexandre Bissoli, Mariana M. Sime, Hilton M. Lessa, Ivan R. Enriquez, Anselmo Frizera-Neto, Teodiano Bastos-Filho

**Affiliations:** 1 Postgraduate Program in Electrical Engineering, Assistive Technology Center (NTA), Federal University of Espirito Santo (UFES), Vitoria 29075-910, Brazil; eduardo.hmc1@gmail.com (E.H.M.-C.); alexandre-bissoli@hotmail.com (A.B.); hilton.lessa@gmail.com (H.M.L.); frizera@ieee.org (A.F.-N.); teodiano.bastos@ufes.br (T.B.-F.); 2 Postgraduate Program in Biotechnology, Assistive Technology Center (NTA-UFES), Federal University of Espirito Santo (UFES), Vitoria 29047-105, Brazil; berthilbl@gmail.com (B.L.); mariana.sime@ufes.br (M.M.S.); 3 Department of Statistics, Federal University of Espirito Santo (UFES), Vitoria 29075-910, Brazil; ivan.guzman@ufes.br

**Keywords:** alternative interface, electric-powered wheelchair, eye tracking, immersion, joystick, simulator, training, virtual reality

## Abstract

For some people with severe physical disabilities, the main assistive device to improve their independence and to enhance overall well-being is an electric-powered wheelchair (EPW). However, there is a necessity to offer users EPW training. In this work, the Simcadrom is introduced, which is a virtual reality simulator for EPW driving learning purposes, testing of driving skills and performance, and testing of input interfaces. This simulator uses a joystick as the main input interface, and a virtual reality head-mounted display. However, it can also be used with an eye-tracker device as an alternative input interface and a projector to display the virtual environment (VE). Sense of presence, and user experience questionnaires were implemented to evaluate this version of the Simcadrom in addition to some statistical tests for performance parameters like: total elapsed time, path following error, and total number of commands. A test protocol was proposed and, considering the overall results, the system proved to simulate, very realistically, the usability, kinematics, and dynamics of a real EPW in a VE. Most subjects were able to improve their EPW driving performance in the training session. Furthermore, all skills learned are feasible to be transferred to a real EPW.

## 1. Introduction

Health conditions like spinal cord injury, traumatic brain injury, multiple sclerosis, quadriplegia, cerebral palsy, stroke, and some congenital problems can lead to movement disorders that significantly reduce quality of life. According to global estimates in 2018, approximately 1% of the world population requires a wheelchair for activities of daily living (ADL) [1]. For some people with severe physical disabilities, the main assistive device to improve their independence in ADL and to enhance overall well-being is an electric-powered wheelchair (EPW).

Since many wheelchair users find nearly impossible to drive with conventional EPW interfaces [2], a wide variety of alternative approaches has been proposed for EPW control [3], including special joysticks [4], EEG (electroencephalography) [5], EMG (electromyography) [6], eye gaze tracking [7], and hybrid EEG/EMG [8]. Additionally, to prevent any safety risks when driving EPW, several algorithms have been developed, including obstacle avoidance technics and autonomous navigation [9]. Most of these types of approaches are part of smart wheelchairs, which are EPW equipped with systems that collect information to modify the wheelchair behavior and could potentially be used for training sessions [10]. 

There is a necessity to offer customization of interfaces to new EPW users and EPW training [11]. However, a major challenge found in [10] was the integration of intelligent features with commercially available EPWs since they are proprietary systems, moreover, the cost of the technology can be a huge barrier. 

The use of systems that provide training in virtual reality (VR) scenarios is becoming a potential tool to support and improve rehabilitation outcomes and physical therapies [12], due to their wide variety of benefits, including safe controlled environments, low cost, and flexibility.

In [13], some evidence of VR training with wheelchair simulators was provided, which can be effective and increase the motivation of the users. The main aspects of wheelchair simulators identified by the authors in [13], are being user-centered, providing different control interfaces, and allowing for a wheelchair behavioral simulation.

There are several VR environment approaches on the literature applied to EPWs whose objectives are concerned with the improvement of driving wheelchair skills, as reported in [14], including the proposal of joysticks to improve virtual EPW driving in individuals with tremor [4], and a virtual wheelchair simulator with hand motion controller as an interface for reaching tasks [15].

**Figure 1:**
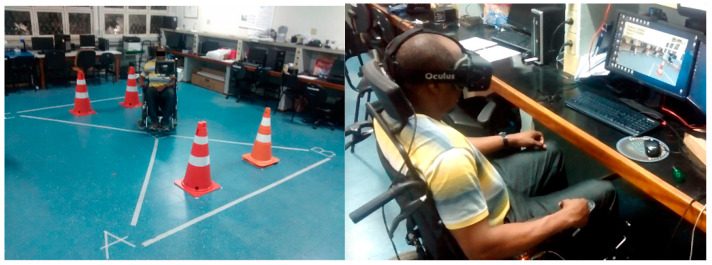
Participant testing the real EPW (**left**) and the virtual one (with the joystick as input interface) in the Simcadrom (**right**).

In this work, the Simcadrom (Portuguese acronym for Simulator of Electric-Powered Wheelchairs) is introduced. It is a VR simulator for EPW driving learning purposes [16], which aids in the customization and test of new functionalities and methods in a safe environment [17], and the testing of control interfaces. Other works have also presented simulators for training EPW that indicate improvement in their users’ driving skills needed for a real EPW [18,19,20]. Similarly to [21], quantitative and qualitative parameters were defined in this work to measure participants’ VR experience and driving performance, and then validate the Simcadrom by comparing between virtual and real tasks [22] from the EPW driving experience in a test protocol (see Figure 1).

Joysticks are the standard control devices for conventional EPWs. Therefore, a proportional standard movement sensing joystick is selected here as a reference and main guidance input interface for the virtual training system. The same can be seen in other wheelchair simulators as reported in [13]. Some researches opt to use regular Microsoft Xbox controllers like in [19] or even adapt them and their position to emulate a real wheelchair joystick controller [20], depending on their application. In this work, a real EPW’s joystick is used to increase realism, as done in [23].

As stated in [10], smart wheelchairs can be multi-modal, multi-functional EPW platforms that can aid in training. Therefore, in addition to the joystick, an eye gaze tracking system, from now on called eye tracker (ET) is integrated as an alternative input interface in a multi-modal control system for our smart wheelchair [24] to command the EPW. 

This work is part of a joint research project between the Federal Universities of Espirito Santo (UFES), Uberlândia (UFU), and Amazonas (UFAM), Brazil [11]. This stage of the project is focused on the development of a safe training system and its technical validation. Future works will focus on end-user groups with motor disabilities.

User perception can be divided into different parts according to the sense that is stimulated, vision, hearing, vestibular, or force feedback [25]. All of them are important and have an impact on the user’s immersion in the simulator. In one of its configurations, the Simcadrom uses VR, a video projector, and a Head-Mounted Display (HMD), which is commonly used in wheelchair simulators as reported in [13]. Sound may also be paired with visual feedback to improve immersion and motivate training [26], thus, our simulator provides auditory feedback to the users about collisions [27] and the EPW motors. 

Even though VR is a great tool for wheelchair simulators, it can produce cybersickness as reported in [28,29], which is a major disadvantage produced basically by perception conflicts when the users see themselves moving in the virtual EPW but they do not feel the physical accelerations from their movements. 

In [23], a mechanical platform that provides vestibular feedback and haptic feedback was added to their simulator system in addition to their visual display showing the VE, and they reported having increased immersion and decreased cybersickness for users. This stage of our research, will not address vestibular feedback since the wheelchair simulator would need to incorporate a mobile platform that increases greatly the price and complexity of the simulator [13]. However, in the “Materials, Methods, and Experiment Setup” section, some measures that were taken to produce a realistic simulation while trying to reduce cybersickness symptoms are described. For instance, providing the users with haptic feedback by having them sitting in a real EPW and using a real EPW’s joystick. 

Moreover, an immersion exploration using a computer monitor, like in [30], was done in [31] with the Simcadrom, where a comparison was done between three displays (HMD, desktop screen, and projector) using IPQ and Simulator Sickness Questionnaire (SSQ) as in [23]. In this work, the simulator aims to offer the maximum immersion possible, using the HMD, or a projector.

Among the different types of studies and their objectives classified in [13], this work fits in the groups where there is a need for realistic virtual wheelchair behavior [32] and where an evaluation of the effectiveness of VR training to improve powered wheelchair use is done [30,33]. The experimental protocol used in this work considers these objectives.

The driving performance quantitative parameters for this simulator consider: time spent executing a given task [22], path following error [34], and the number of movement commands made with the input interface [21]. These real-time recorded data require a more sophisticated level of instrumentation [13] since the data is used to compare a virtual EPW with a real one. This is considered a major contribution of this work, for the best of the author’s knowledge. It seems like most of the related works do not have a test protocol that uses as many quantitative measurement parameters for comparison purposes with a real EPW, perhaps to avoid the complexity associated with instrumenting an EPW.

This work also contributes with a novel approach to evaluate and classify the number of movement commands for continuous input interfaces to assess possible changes throughout training with that input interface. An alternative input interface like the eye tracker generates a discrete number of movement commands, thus, a command classification method to get the number of commands made with a joystick is proposed. Therefore, both type of interfaces can be measured using the same metric.

In addition to the quantitative data, the qualitative part of the simulator evaluation contemplates subjective user experience questions that participants answer about their experience using the Simcadrom (see Section 4.8). The Igroup Presence Questionnaire (IPQ) [35] was used here to measure participants’ sense of presence in the VE, as in [15]. These are commonly used methods to provide information about the impact of realistic graphics, behaviors, or immersion [13]. 

## 2. Hypotheses

Simcadrom was developed as a training system for learning how to control an EPW using a joystick or an alternative input interface such as an eye tracker. Thus, three hypotheses were established and tested:

- Simcadrom simulates a real EPW close enough so it can be used for virtual training.
- Subjects of the test protocol can learn and improve their EPW driving skills by doing a training session in the Simcadrom.
- Skills learned in the training session in the Simcadrom can be transferred to the real EPW.

A test protocol was designed to test these hypotheses. Transference of skills evaluation was based on the methods proposed in [36,37,38].

## 3. Materials, Methods, and Experiment Setup

The user sat in a real EPW while using the Simcadrom to increase the realism of the virtual driving experience and for haptic feedback purposes. However, no movement was induced to this EPW (from Freedom, Brazil) and thus no proprioceptive or vestibular feedback was given to the user.

### 3.1. Virtual Environment of the Simcadrom

**Figure 2:**
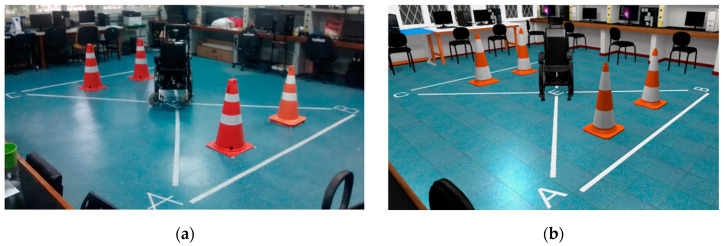
Real (**a**) and virtual (**b**) NTA Research Group Laboratory.

In contrast with other studies where wheelchair simulators do not try to reproduce reality with their VEs [20], or they reproduce scenarios that participants were not necessarily familiar with [19,23], the VE for this study attempts to recreate a realistic scenario and behavioral simulation of the virtual EPW close to the behaviors of the real one and its environment. It was of the authors’ interest to evaluate the system with the least disturbing variables involved. Therefore, a virtual model of the NTA Research Group Laboratory (Assistive Technology Center at UFES/Brazil) was developed for comparison purposes with the actual real environment, shown in Figure 2. If the user looks down, they will see virtual legs corresponding with their real legs, as it was done in [20].

**Figure 3:**
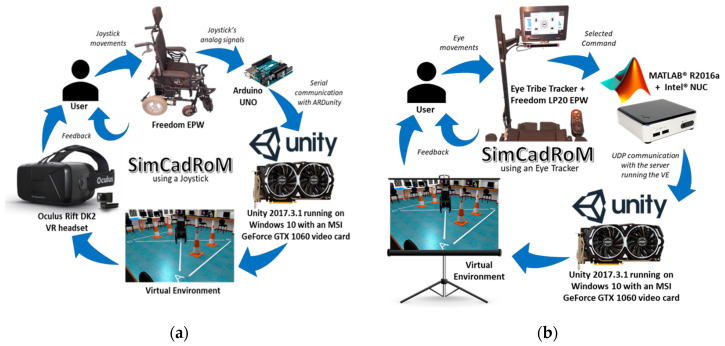
Main components of the Simcadrom when using the joystick (**a**) or the eye tracker (**b**).

The selected VE was created with Unity 3D game engine and runs on a PC equipped with Windows 10, 8 GB of RAM, Intel i7 processor, and an MSI GeForce GTX 1060 graphics card. The other main components of the simulator, when using a joystick or the eye tracker, are shown in Figure 3a,b respectively.

The simulation system with a joystick as the input interface uses the actual joystick of the real EPW as the input interface for the virtual EPW. An Arduino UNO board attached to the wheelchair acquires analog voltage signals from the joystick and sends them to the VE in Unity running on the PC, by USB serial communication using ARDunity Basics libraries.

Finally, the system is equipped with an HMD to show the user the VE. The device selected was the Oculus Rifts DK2 VR headset, which the user wears while sitting in the real EPW and using its actual joystick. Thus, it offers a greater immersive VR driving experience, controlling the virtual EPW.

**Figure 4:**
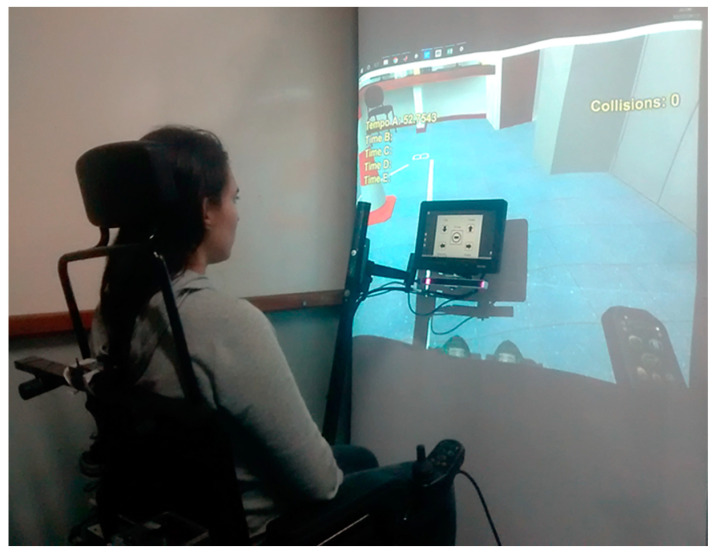
A participant using the Simcadrom with an eye-tracking device as the input interface.

For an alternative interface like the eye tracker, the simulation system does not make use of the selected HMD to show the VE to the user and, at the same time, do eye gaze tracking, at least not with that VR headset model. Therefore, a practical solution for still providing near real-size visual feedback and an immersive experience was to use a projector and a screen as shown in Figure 4, while the user sits on the real EPW with no movement. The real EPW, shown in Figure 4, was equipped with an LCD screen that displays a navigation software interface for the users to look at and select the direction in which they want the EPW to move or turn and a resting area in the middle with no functionality [24].

### 3.2. The Velocity of the EPW Using a Joystick

The eye tracker interface sets discrete velocities references for the EPW. On the other hand, proportional joysticks are commonly used to change EPW’s velocity in proportion to the amount of deflection (typically 0°–18°) of the spring-loaded joystick post. Thus, the wheelchair moves approximately in the direction the handle is pointed. Additionally, features such as dead zone, gain, and axes rotation are also mechanically defined. 

Considering all these characteristics, some tests were conducted to acquire the analog voltage signals from the real EPW’s joystick and its mechanical deflections, to later emulate those signals and obtain various EPW’s velocities, which were restricted to comfortable levels to try to reduce cybersickness, as recommended in [39]. A dynamic model of the EPW was not calculated in this project, however, the obtained velocities were registered for different loads, since the linear and angular velocities of the real EPW are affected by the wheelchair user’s mass (the position where the user sits was not considered).

**Figure 5:**
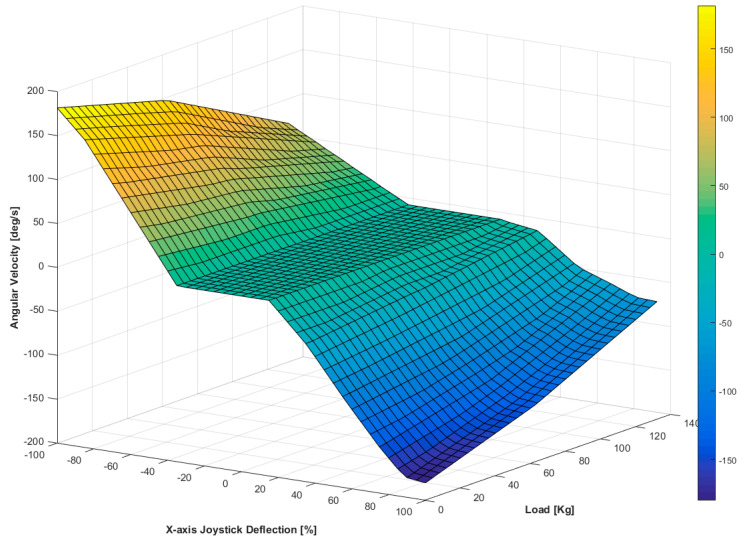
Experimental angular velocities of the real EPW for different mass and different joystick’s *X*-axis deflections.

The obtained data were interpolated in three dimensions to get a closer approximation of the angular and linear velocity of the real EPW considering dynamic effects, thus, representing better its behavior in the VE. Figure 5 shows an example of how the interpolated velocities of the EPW look.

### 3.3. Obtention of the Path Following Error

The participants were asked to drive the EPW over the areas marked by the letters starting at point E, in the middle of the NTA Research Group Laboratory, and then passing through points A, B, C, D and then returning to E, as indicated by the marks on the floor shown in Figure 2.

The participants were instructed orally in their native language to drive the real and virtual EPW over the path without hitting the cones by following the reference path and to complete it as quickly and as accurately as possible. The movements from the real EPW were measured by encoders and an IMU (FRDM-FXS-MULTI-B) [40].

**Figure 6:**
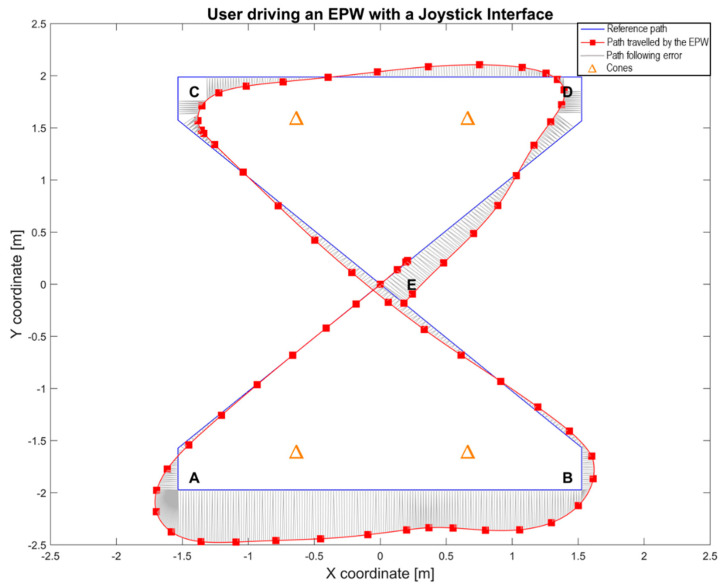
The path followed by the real EPW using a joystick.

Data of one of the trials from a participant using a joystick is shown in Figure 6, where the reference path and the traveled path by the real EPW are presented. This figure also shows error lines, calculated as Euclidean distances, coming from the traveled path to the nearest point in the corresponding segment of the reference path.

**Figure 7:**
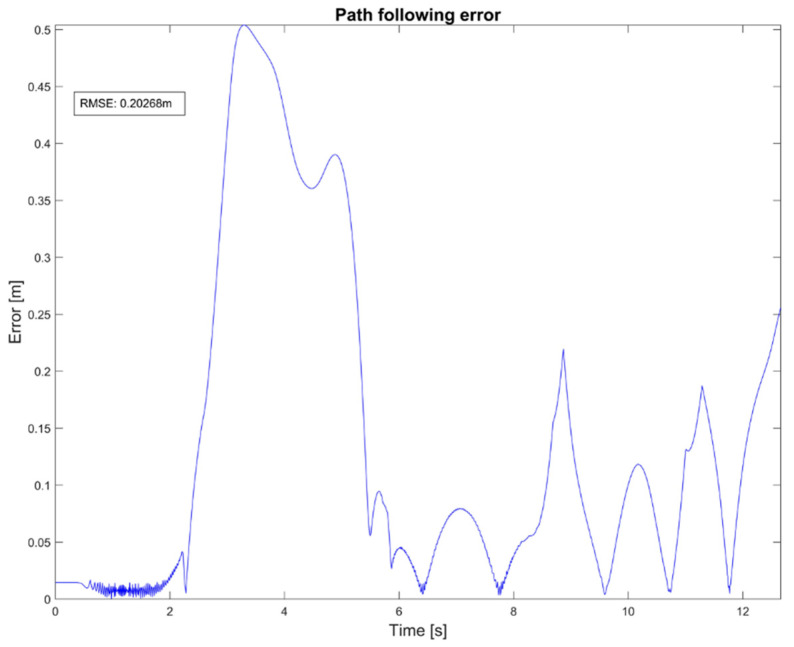
Path following error from the real EPW using a joystick.

Every calculated distance was then considered as an amplitude error, which was registered in a time series to produce an error signal as the one presented in Figure 7. Consequently, the root-mean-square error (RMSE) was obtained from this signal and registered for every participant using a joystick in the VE and the RE.

### 3.4. Obtention of the Total Number of Commands

In this work, two different input interfaces that command the EPW are used and, although they cannot be compared directly because of their different characteristics, they are measured using the same metric, which is the number of commands required by the participant to drive the EPW using those interfaces. Then, the total number of commands and the way it is obtained here is useful for the next input interfaces that can be added to the simulation system.

The commands generated by the eye tracker interface can be counted easily as it generates discrete commands over time for turning left or right and for forward or backward when the user looks constantly for a specific period of time at a direction arrow on the user interface. For safety reasons, the EPW moves forward or backward a specific preprogrammed distance when the correspondent discrete command is selected. It also rotates clockwise or counterclockwise a fixed preprogrammed amount of degrees, depending on which turning command was selected. These commands go to the VE (in the case where the user is performing tests in the VE) or to the real EPW controller (for the RE tests with the eye-tracker). In the case of the RE, these commands go to a PID controller empirically tuned in a closed loop aiming to get a smooth and slow control action that does not cause angular position overshoots in the EPW.

**Figure 8:**
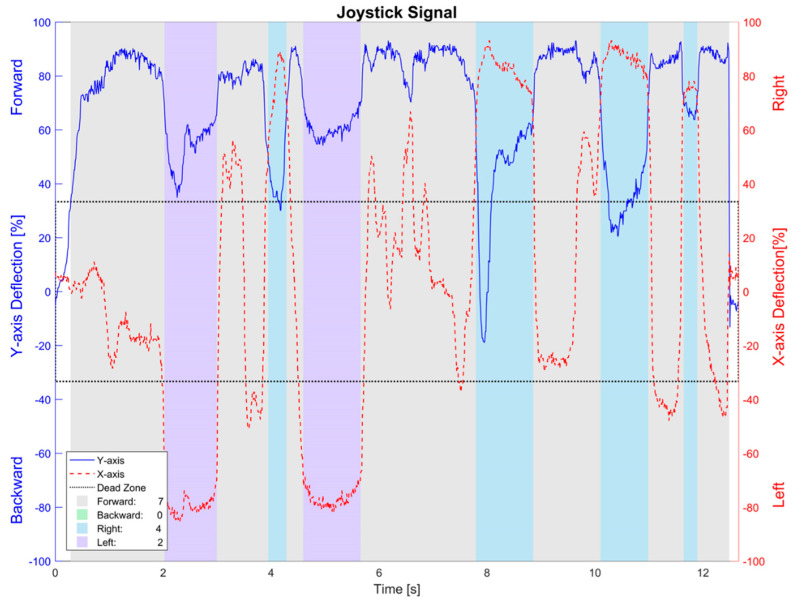
Classification of commands from joystick’s signals while driving the real EPW.

Identifying an individual movement command is not that evident in the case of an input interface like a joystick, as it generates analog voltage signals in X and Y axes, respectively (shown in Figure 8 as red-dashed and blue-constant signals). This is even less evident considering the dead zone the real EPW has, for safety reasons. 

Therefore, it is proposed here to implement a classifying algorithm of the samples from the joystick’s signals along the elapsed time during the tests. The X and Y-axes signals are considered to be in rectangular coordinates, which are then converted to polar coordinates to get their magnitude and angle/direction of the joystick. 

Afterward, this is used to detect if a sample belongs to what is going to be considered as a movement command, meaning that its magnitude is above the dead zone threshold, and then specifying whether it is a forward, backward, right, or left command, depending on its angle.

The beginning of the first command is detected by its magnitude, and the end of it is considered to be when the command has changed to another one or when no command is detected (below the dead zone threshold). Finally, the total number of commands during the trial can be counted.

### 3.5. Experiment Setup and Participants Selection

The simulator was evaluated qualitatively by both a user experience questionnaire (UEQ) and the IPQ, and quantitatively by doing statistical test comparisons between participant’s driving performance, through the following parameters: time spent executing a given task, path following error, and the number of movement commands made with the input interface. The comparisons to evaluate the established hypotheses were included in a test protocol designed for the training session with the Simcadrom using the joystick and eye tracker. 

It is reported in [10] that there is a “lack of standardization in assessment and training protocols” for EPWs, however, there are a few tools highlighted in the literature [41]. It is also mentioned that, depending on the context, the responsibility to best determine the evaluation approach lies in the EPW provider. In our case, the test protocol designed for the experiments does not follow an established training wheelchair protocol like the ones mentioned in [41] or [42]. Also, in contrast with the works mentioned above, our study reports first trials for wheelchair training. Future works of our research involve the proposal of a new training protocol, which will be based on the experience acquired from the several experiments we have conducted.

This study, which involves VR, was approved by the Federal University of Espirito Santo Ethics Committee (protocol number 2264126), and constitutes a mandatory step before clinical trials and, as such, only enrolled people without any physical impairment, who do not represent the demographics of real EPW end users. Twenty healthy unpaid participants were recruited to this study. Ten of them used the joystick as the simulator’s input interface during the tests, and the other ten used the eye tracker as an alternative interface. 

Participants were between 18 and 36 years old and weighed between 45 and 120 Kg. Inclusion criteria consisted of having a normal or corrected vision, and for the case of the group that used the joystick, the participants were right-handed and had their right arm and right hand able to control the EPW with its joystick. These participants also required proprioception and dexterity at joints to efficiently use the proportional control, such as done in [43]. The participants were asked if they could perform the tests on both RE and VE and were told they could leave at any time. 

**Figure 9:**
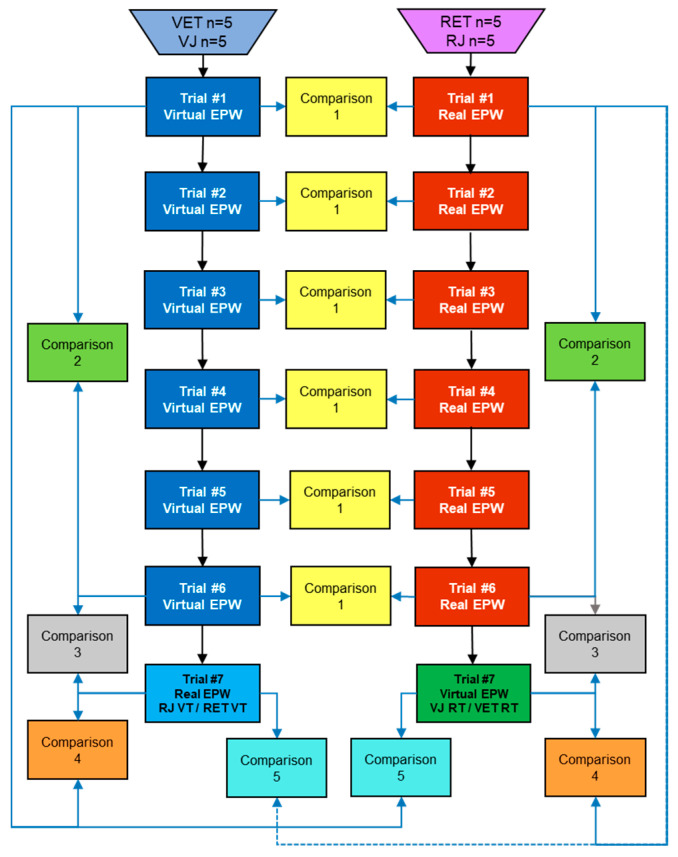
Proposed test protocol for all the trials and comparisons.

The 20 participants were divided into four groups (n = 5) as presented in the test protocol in Figure 9, where one group used the joystick and trained in the VE, named “VJ” (virtual joystick). The second one is called “RJ” because they used the joystick and trained in the real EPW. The third one used the eye tracker and trained in the VE, called “VET” (virtual eye tracker). The last group is “RET”, who used the eye tracker and trained in the real EPW. The participants were not given any previous training for the tests, they received basic instructions before their first trial and their elapsed time throughout the session as additional feedback.

Homogeneity is assumed, since the following characteristics, that are being considered in this study as relevant, are presumed to affect each group’s performance in the same proportion:

- The groups of participants “VJ” and “RJ” had very similar mean weight (81.4 ± 15.12 Kg and 80.6 ± 15.73 Kg), as well as the “VET”/“RET” groups (65.6 ± 10.25 Kg and 67.0 ± 15.47 Kg).
- None of the participants needed an EPW or had driven one in the previous year.
- Two out of five participants from each “VJ” and “RJ” groups had used an HMD like Oculus Rift before, at least once in the previous year.
- Two out of five participants from each “VET” and “RET” groups had used the eye tracker before, at least once in the previous year.
- Four out of five participants from each “VJ”, “RJ”, “VET” and “RET” groups played “First-Person Shooter” games before, at least once in the previous year.
- One out of five participants from the “VJ”, “RJ” and “VET” groups did not know how to drive a motor vehicle.

### 3.6. Test Protocol

The participants from the “VJ” and “VET” groups were asked for the training session, to drive the virtual EPW following the specified reference path six times, such as done by [44], with less than five minutes between trials. Then, for the seventh trial (the final test for their training), they were asked to drive the real EPW in the RE. This same procedure was done by the “RJ” and “RET” groups, beginning in the RE, with the final test in the VE, for counterbalancing the influence the order of the tests could have on the results.

### 3.7. Comparisons in the Test Protocol

The data obtained from the measurements of each group of participants were considered to be different for each of their respective trials since a learning effect was presumed, i.e., data from the first trial of one of the four specified groups of participants should not be assumed to belong to the same measurements from the second trial of the same group of people. Since the data from each group of participants are divided by their trials, a comparison, named “Comparison 1”, between “VJ” and “RJ”, or “VET” and “RET”, was done between each trial with its correspondent to aid in finding how close the virtual experience is to the real one (Hypothesis 1). 

Once the “VJ” and “VET” groups finished the six trials in the VE and the “RJ”, and “RET” groups finished their final trial in the VE, the participants were asked to fulfill the IPQ test in Portuguese (instrument properly validated in different cultural contexts) [45], to measure their sense of presence in the VE. And after they all finished the whole test (seven trials), they were asked to answer the UEQ (5-point Likert scale) about their experience using the Simcadrom. These questionnaires also helped in finding how close the virtual experience was to the real one (Hypothesis 1). 

In order to evaluate the other hypothesis, about people learning by doing a training session (Hypothesis 2) and transferring the acquired knowledge to the opposite environment (Hypothesis 3), some comparisons were considered into the protocol. “Comparison 2” helps to measure if there was any learning effect by calculating whether measurements from trial “six” from a group of participants were significantly better than the ones from trial “one” of the same group (Hypothesis 2). 

Presuming that people learned in the training session and that the VE is similar to the RE, “Comparison 3” helps in finding out if the acquired skills of a group of participants after trial “six” are maintained or improved in trial “seven” in the other environment (Hypothesis 2 and 3). “Comparison 4” is also based on the previous assumptions and tells if the learning effect from trial “one” is extended until trial “seven” (Hypotheses 2 and 3). 

Finally, “Comparison 5” estimates how good were the measurements from trial “seven”, in contrast with the measurements from the first trial of the opposite environment (Hypothesis 3). In other words, these final measurements, that were obtained from the trial in an environment that is the opposite to the one where the group trained, are compared to the measurements from the first trial of the other group of people in that same environment, but with no training. For instance, trial “seven” of the “VJ” group, also named “RJ VT” (from RE using Joystick, after Virtual Training), was compared to trial one of the “RJ” group. These comparisons aid in proving/disproving quantitatively the hypotheses, however, qualitative data must also be considered.

## 4. Results and Discussion

In this section, the qualitative and quantitative results from the abovementioned protocol test are analyzed in order to assess whether they support the hypotheses stated before (readers may find all the data in the tables in the Supplementary Materials).

The following subsections present the statistical analysis from the measurements obtained. These subsections are divided in: elapsed time, path following error, and the number of commands, for both VE and RE. Thereafter, the data from the questionnaires are presented. In some cases, more than one dependent or independent statistical test of the difference of two means was conducted for the same data set. Therefore, the probability of making type I errors (family-wise error rates) while performing multiple hypotheses tests may increase. 

Some methods have been proposed to circumvent the problem, due that as the number of tests increases, so does the likelihood of a type I error. The Bonferroni correction is one of the most popular methods widely used in various experimental contexts, as it adjusts probability (*p*) values because of the increased risk of having falsely rejecting null hypotheses when making multiple statistical t-tests. However, this method has been criticized for testing the wrong hypothesis, and for reducing the chance of a type I error, but at the expense of a type II error [46].

Thus, one of the limitations of this investigation is that the increase in the family-wise error rate across the reported statistical analyses was not controlled [47]. Overall, we consider this research relatively preliminary and encourage replication. 

### 4.1. Time Analysis When Using a Joystick

The elapsed time to drive the EPW along the reference path in the RE and VE was defined as the first quantitative parameter to compare driving performance, which was registered per trial for each participant. The mean of the total elapsed times and their standard deviations were calculated for every trial of each of the four groups (n = 5): “VJ”, “RJ”, “VET”, and “RET”. Since the test protocol does not intend to infer the behavior of the entire end-user population based on its results, a population standard deviation was implemented, which considers the test subjects as its entire population.

These values were compared between trials to see whether their difference was statistically significant or not, see Table 1.

As we are interested in the mean values, and the sample number was small (n = 5), a Shapiro-Wilk test was conducted to see if those differences had a normal distribution. This test was reported in [48] as one of the best normality tests since it rejects the null hypothesis of normality at the smallest sample size compared to the other tests, for all levels of skewness and kurtosis of these distributions. The test showed that those differences can be assumed to have a normal distribution, since a *p*-value greater than a level of confidence of 0.01 was obtained for the six trials.

After the normal distribution condition was assumed to be met, a Fisher’s F-test of equality of variances was performed to find out whether the data’s variance from each pair of trials from the RE and VE could be considered equal or unequal. This way, the proper t-test to apply could be better determined.

Finally, an unpaired t-test, assuming equal variances, showed enough evidence to accept the null hypothesis (*p* > 0.05). There is no statistically significant difference between the data obtained from the total elapsed times of each pair of trials during the training sessions in the VE and RE. This same procedure was performed for the other criteria (path following error and number of commands) for both interface uses (joystick and eye tracker), in both virtual and real training environments.

**Figure 10:**
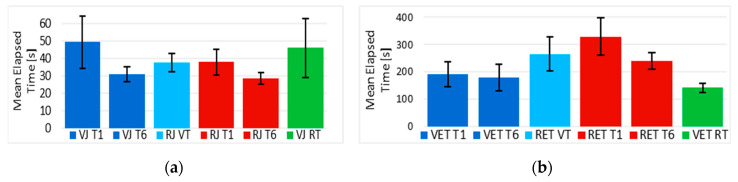
(**a**) Mean elapsed times of trials 1, 6, and 7 from the VE and RE using a joystick; (**b**) Mean elapsed times of trials 1, 6, and 7 from the VE and RE using eye tracker.

Following, Figure 10a displays the mean elapsed times from the first, sixth, and seventh (final) trials of the training session with a joystick in the VE and RE. The first trial in the VE (“VJ T1”) and RE (“RJ T1”) are measurements of performance without any previous training, using time as a parameter. “VJ T6” and “RJ T6” are the times at the end of the training session for the “VJ” and RJ” groups, respectively. These times decreased after, and their variability decreased as well.

“RJ VT” (RE with joystick after virtual training) seemed greater in time than “VJ T6”, lower than “VJ T1”, and about the same than “RJ T1”. A very similar thing happened to “VJ RT” (VE with joystick after real training), seemed greater than “RJ T1” and “RJ T6”, and almost equal to “VJ T1”, even in variability. However, in order to conduct a more precise analysis, Table 2 shows a paired one-tailed t-test that was applied between the first and sixth trials of the VE and also in the RE (“Comparison 2”).

The results rejected the null hypothesis (*p* < 0.05), indicating that the elapsed times from the first trial (no training) were significantly greater than the ones after the training session of the same groups of participants.

In “Comparison 3”, a paired two-tailed t-test was done for each group between the times acquired in trial “six” and the ones from the final trial, to see if the learned skills of a group of participants after trial “six”, somehow were maintained in trial “seven” in the other environment. Results revealed that there was a significant difference between times from “VJ T6” and “RJ VT” (*p* < 0.05), but the null hypothesis could not be rejected for “RJ T6” and “VJ RT” (*p* > 0.05), which means that there is not enough evidence to say that the elapsed times of “VJ RT” were significantly different than those of “RJ T6”, suggesting, in this case, the same level of performance in the opposite environment.

“Comparison 4”, which is also based on the learning effect assumption, compares if measurements from trial “one” are significantly greater than those of trial seven with a paired one-tailed t-test. Results showed that after the virtual training, the obtained mean elapsed time from the “RJ VT” was significantly smaller (*p* < 0.05) than the time from “VJ T1”, as expected. Nonetheless, this was not the case in the opposite order, where the time from “RJ T1” was not significantly different than the time from “VJ RT”, suggesting not only that the same level of performance was maintained in the opposite environment, but that there was not enough evidence that the improvement in time continued in the VE.

Finally, “Comparison 5” estimated that the measurements from trial “seven”, in contrast with the measurements from the first trial in the opposite environment, had no significant difference (*p* > 0.05) by implementing an unpaired one-tailed t-test, whose variances were assumed equal or unequal depending on F-test *p*-values at the 0.05% level. In the case of the times obtained by “RJ VT” (the group of participants using a joystick in a real EPW for the first time after training in the VE), their variance was considered to be equal (F(4,4) = 2.04) to the one of “RJ T1”. And for “VJ RT”, their variance was unequal (F(4,4) = 1.28) to the variance of the “VJ T1” groups. Thus, there is not enough evidence to see significant progress reducing the elapsed times after a training session in the VE nor RE.

### 4.2. Time Analysis When Using Eye Tracker

Following the reference path in the tests using the eye-tracker interface with its driving modality, defined above, took more time than driving the EPW with a joystick, as it was expected.

“Comparison 1” of these normally-distributed data (*p* > 0.05) revealed with an unpaired t-test that there is no statistically significant difference (*p* > 0.05) between the mean of the total elapsed times during the training session in VE and RE using eye tracker in trials 2, 5, and 6. However, the null hypothesis was rejected (*p* < 0.05) for trials 1, 3, and 4.

It can be said, from “comparison 2”, that there was no significant difference (t(3) = 0.779, *p* = 0.246) between mean times from “VET T1” and “VET T6” shown in Figure 10b. However, when training in the RE, the times from “RET T1” were significantly greater (t(4) = 2.326, *p* = 0.04) than those of “RET T6” after training.

“Comparison 3” showed that there is a significant difference (*p* < 0.05) between “VET T6” and “RET VT”, and between “RET T6” and “VET RT”. This suggests that, at least for the time parameter, the performance was not maintained when a group of participants trained with eye tracker in the VE. However, the improvement of the training in the RE was maintained in the virtual one.

Furthermore, it was obtained from “Comparison 4” that the mean time in “RET VT” was significantly greater (t(3) = 3.233, *p* = 0.024) than the mean time in “VET T1”. However, times from “RET T1” are on average greater (t(4) = 5.777, *p* = 0.002) than the ones from “VET RT”. Then, “Comparison 4” confirmed the same behavior seen with “Comparison 2”, in which there was an improvement of times just when training with the eye tracker in the RE but not in the VE, and this improvement continues in the opposite environment. This suggests that driving the EPW with the eye tracker in the VE was somehow easier than driving the real EPW with the same interface.

**Figure 11:**
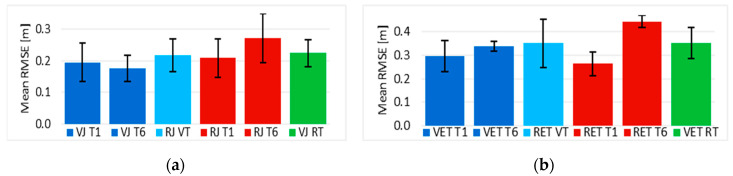
(**a**) Path following error of trials 1, 6, and 7 from the VE and RE using a joystick; (**b**) Path following error of trials 1, 6, and 7 from the VE and RE using eye tracker.

Although a difference can be noticed between the mean times obtained in “RET VT” (RE after training in the VE) and “RET T1” (RE with no previous training) in Figure 11, results from “comparison 5” showed there was no significant difference (t(8) = 1.404, *p* = 0.099) between them.

However, the mean time from “VET T1” was significantly greater (t(7) = 2.025, *p* = 0.041) than the time from “VET RT”, which suggests more evidently that the training in the RE with eye tracker helped participants from the RET group to perform better in the VE than the VET group.

### 4.3. Path Following Error Using a Joystick

A mean of the RMSE values was calculated for the “VJ” and “RJ” groups per trial. The statistical tests indicate that the differences between the mean values per trial can all be assumed to have a normal distribution (*p* > 0.02) and that there is enough evidence to say there were no significant differences in RMSE values between the trials from the VE and the RE using a joystick (*p* > 0.05), except for trial “five”, where the null hypothesis was rejected (t(7) = 3.213, *p* = 0.015). This suggests that the path following error was similar between the VE and RE using a joystick (“Comparison 1”).

The statistical tests for the other comparisons, based on the data shown in Figure 11a, revealed that there was no improvement (“Comparison 2”) of the path following error during the training using a joystick, since there was no significant difference (*p* > 0.05) between none of the mean values per trials in the VE nor RE. Although an error increment in “RJ T6” can be evidenced, it is not significant (t(3) = 0.904, *p* = 0.216). This also means for “comparison 3” that regarding the path following error parameter, the performance was at least maintained in the opposite environment of the training session using the joystick.

### 4.4. Path Following Error Using Eye Tracker

In a similar way to the case of the joystick interface, the differences between the RMSE mean values per trial using eye tracker can all be assumed to have a normal distribution (*p* > 0.02). Also, there is enough evidence to say there were no significant differences in RMSE values between the trials from the VE and the RE (“Comparison 1”) using eye tracker (*p* > 0.05), except for trial “six”, where the null hypothesis was rejected (t(6) = 5.491, *p* = 0.002). A deeper analysis was done, based on the values shown in Figure 11b.

The statistical tests for “Comparison 2” show that there was no improvement of the path following error during the training in the VE or RE (*p* > 0.05), but interestingly, their variabilities were reduced. “Comparison 4” indicated that there was no improvement of the path following error after the training in the VE or the RE (*p* > 0.05), in fact, it revealed that the RMSE mean value in “RET T6” and “VET RT” was significantly greater (*p* < 0.05) than the first trial “RET T1”. Nevertheless, the performance level of this parameter in “RET VT” after the training in the opposite environment was consistent with “VET T1”. Moreover, there was no significant difference (*p* > 0.05) between the sixth and final trials (“Comparison 3”), suggesting that the performance of the group of participants was maintained in the opposite environment.

Finally, there was no significant difference (*p* > 0.05) of the path following error measurements between driving for the first time the EPW with no training, and driving the same EPW for the first time with previous training in the opposite environment (“Comparison 5”), i.e., between “RET T1” and “RET VT”, and between “VET T1” and “VET RT”.

### 4.5. Number of Commands Using a Joystick

The differences between the number of commands extracted from the “VJ” and “RJ” analog signal curves are considered to be normally distributed (*p* > 0.05). Furthermore, “VJ” and “RJ” were significantly statistically different (*p* < 0.05) when compared between trials, except for trial “one” and “two”, where the null hypothesis was accepted (*p* > 0.05). This “Comparison 1” indicates that most of the training driving the EPW in the VE using a joystick required more commands than driving it in the RE.

**Figure 12:**
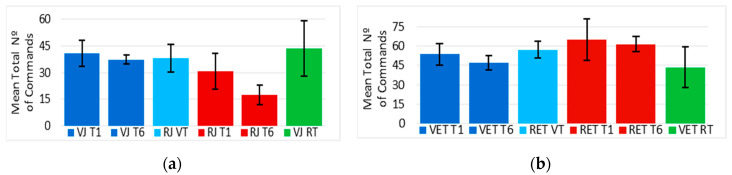
(**a**) Mean total number of commands made in trials 1, 6, and 7 from the VE and RE using a joystick; (**b**) Mean total number of commands made in trials 1, 6, and 7 from the VE and RE using eye tracker.

Additionally, when comparing trial “one” with trial “six” (“Comparison 2”), Figure 12a suggests no improvement of their mean values, no difference was found (*p* > 0.05) for the “VJ” group but a reduction in their standard deviation can be noticed. No significant difference (*p* > 0.05) was found between the number of commands made in trials “seven” and “one” (“Comparison 4”), suggesting that although there was no improvement in performance, at least it was maintained in the opposite environment, which is also supported by “Comparison 3” since there was no significant difference (*p* > 0.05) between “VJ T6” and “RJ VT” in the RE after virtual training. The number of commands in “RJ VT” also did not present a significant difference (*p* > 0.05) with the first trial in the RE “RJ T1” (“comparison 5”).

In the case of the “RJ” group training in the RE, the number of commands in “RJ T1” was significantly greater (*p* < 0.05) than “RJ T6”, suggesting a training improvement (“comparison 2”) in the number of commands required. However, the first trial in the VE (“VJ RT”) of the same group of participants had no significant difference (*p* > 0.05) in the total number of commands with “VJ T1” (“Comparison 5”) nor “RJ T1” (“Comparison 4”). Moreover, the number of commands in “VJ RT” was significantly greater (t(3) = 3.349, *p* = 0.044) than the one in “RJ T6” (“Comparison 3”).

The number of commands made by the “RJ” group using a joystick followed a similar behavior as their elapsed times, clearly showing a relation between them.

### 4.6. Number of Commands Using Eye Tracker

The differences between the “VET” and “RET” commands along the time are considered to be normally distributed (*p* > 0.05). Moreover, “VET” and “RET” were not significantly statistically different (*p* > 0.05) when the means of the total number of commands were compared between trials (“Comparison 1”), except for trial “six”, where the null hypothesis was rejected (t(6) = 3.06, *p* = 0.022).

This indicates that most of the training driving the EPW in the VE using eye tracker required the same amount of commands as driving it in the RE.

Additionally, Figure 12b shows there was an improvement in the commands made during the virtual training (“Comparison 2”), since “VET T1” was significantly greater (t(3) = 2.858, *p* = 0.032) than “VET T6”. There was no difference (t(3) = 0.509, *p* = 0.323) between “RET T1” and “RET T6” in the RE, suggesting that there was no training, since the “Comparison 2” compares only the first and sixth trials. However, the variability was reduced and the first five trials in the RE were not significantly different than the trials in the VE, in which an improvement in commands could be demonstrated.

It can also be said from this figure that the commands in “RET VT” had no difference (*p* > 0.05) with “VET T1” (“Comparison 4”) nor “RET T1” (“Comparison 5”), but the data became more homogeneous. Besides, when “RET VT” was compared to “VET T6” (“Comparison 3”), the null hypothesis was not rejected (t(3) = 3.108, *p* = 0.053). This could indicate that the performance measured with the number of commands was maintained in the RE and that the simulator required fewer commands than the RE when using eye tracker, since there was an improvement during the training in the VE, which was also manifested in the reduction of variability in “RET VT” compared to “RET T1”.

“RET T6” and “RET T1” were significantly greater (*p* < 0.05) than “VET RT” (“Comparison 3” and “Comparison 4”), indicating an improvement in the VE. Furthermore, “VET T1” was also significantly greater (t(8) = 2.25, *p* = 0.027) than “VET RT” (“Comparison 5”), suggesting that the training in the RE helped the “RET” group to get the task done in the VE with fewer commands than the “VET” group in their first trial.

### 4.7. Sense of Presence Questionnaire IPQ

Right after finishing the task in the VE, the participants answered the questions from the IPQ. The items used in the survey split into distinct factors: general presence (G1), as the highest-loading item in the IPQ; spatial presence (SP), emphasizing the importance of actions in the VE; evaluations of the interaction or involvement (INV) as a manifestation of the attention component of the presence experience; judgments of realness (REAL) [35] as a comparison between driving the virtual EPW and the real one. All items are rated from 0 to 6; the greater the score, the greater the overall sense of presence using the Simcadrom.

#### 4.7.1. IPQ When Using an HMD and a Joystick

**Figure 13:**
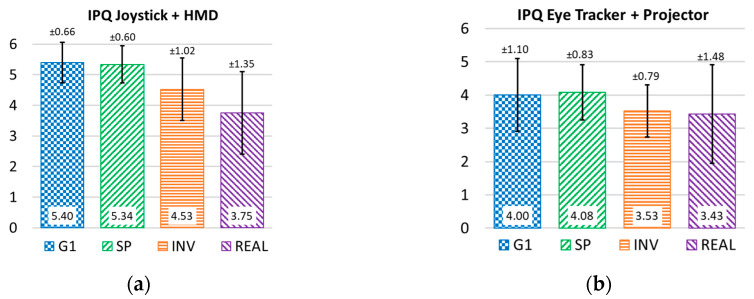
Mean and standard deviation of each IPQ factor after driving the virtual EPW using an HMD and a joystick (**a**), and a projector and eye tracker (**b**).

Most IPQ factors and most of their items show satisfactory results (mean above 4 in a 0 to 6 scale), in Figure 13a, when using an HMD and a joystick.

#### 4.7.2. IPQ Using a Projector and Eye Tracker

When using a projector and eye tracker, the simulation system did not provide a sense of presence score as high as when it used an HMD and a joystick (more details in [31]).

However, it still obtained satisfactory results, since the mean values of its general sense of presence (G1) and spatial presence (SP) factors were higher than four, and the involvement (INV) and realness (REAL) factors above three points, see Figure 13b.

### 4.8. User Experience Questionnaire

**Figure 14:**
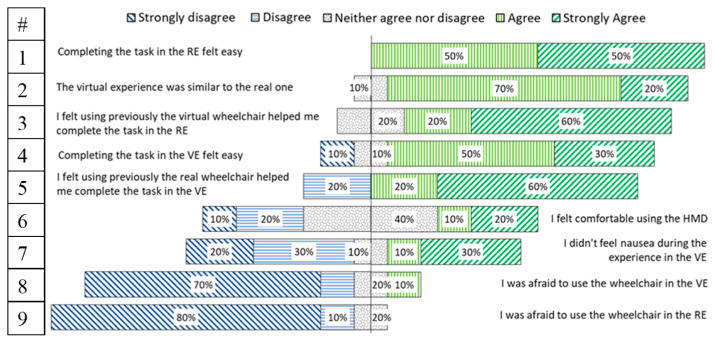
Results of participants’ agreement-disagreement level for each user experience question for the groups that used a joystick in the VE and RE.

**Figure 15:**
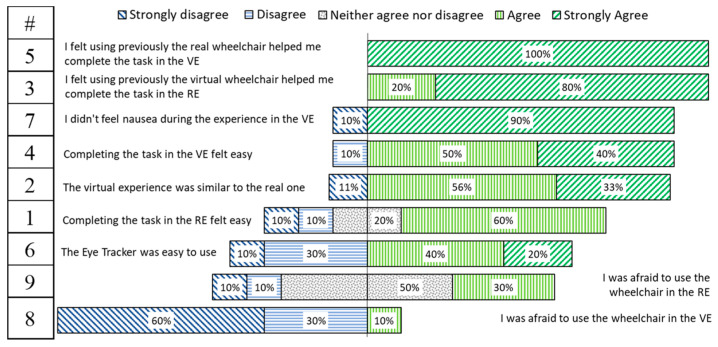
Results of participants’ agreement-disagreement level for each user experience question for the groups that used eye tracker in the VE and RE.

After completing the IPQ, a user experience questionnaire of nine questions was performed, in which the participants had to specify their level of agreement or disagreement on a symmetric agree-disagree 5-point Likert scale. Results are shown in a diverging stacked bar chart, such as suggested by [49]. Figure 14 shows the results for the “VJ” and “RJ”, and Figure 15 for the “VET” and “RET” groups.

These user experience questions showed satisfactory results in users’ acceptance of the Simcadrom as an EPW simulator for training, since participants reported that the experience in the VE felt similar to the real one, which is consistent with the IPQ results. In fact, they felt that using previously the virtual EPW helped them to complete the task in the RE; and most of them felt that completing the task in the VE was easy. On the other hand, thanks to the counterbalancing experiment design, it was noticed by participants that completing the task in the RE felt easier using a joystick than using the eye-tracker interface.

## 5. Conclusions

This work presented the Simcadrom, a simulation system developed to command a virtual electric-powered wheelchair (EPW) for driving training purposes and testing of input interfaces for people with different types of impairments that may be able to use a joystick or an eye tracker. Although the set up for the tests of this study was controlled and done with people without impairments, this research reports first user trials, preparing subsequent more realistic experiments with end users.

A sense of presence questionnaire (IPQ), a user experience questionnaire (UEQ), and some statistical tests for performance parameters like: total elapsed time, path following error, and total number of commands were implemented. All of them were used to evaluate this version of the Simcadrom as a reliable simulator capable of providing a VE very similar to reality. Most subjects were able to learn and improve their skills by driving a virtual EPW while training in the simulator. Afterward, some comparisons included in our test protocol, were made to aid with quantitative data in the discussion of the three hypotheses that were established for this stage of our research. It is worth mentioning that the selected performance parameters (elapsed time, path following error, and number of commands) are not totally independent of each other and that all qualitative and quantitative results are considered in the conclusions.

**Hypothesis** **1.** *Virtual Experience Similar to Reality.*

The simulator satisfactorily represented the kinematics of the real EPW since statistical tests between each pair of trials from the training sessions in the VE and RE (“Comparison 1” presented in the results and discussion section) indicated there was no statistically significant difference (*p* > 0.05) between most of the means of the total elapsed times, the means of the path following errors (RMSE), and number of commands.

The UEQ showed satisfactory results in users’ acceptance of the Simcadrom as an EPW simulator for training, since participants reported that none of them felt afraid of using the EPW in the VE. Actually, most of them felt that completing the task in the VE was easy, and the experience in the VE felt similar to the real one. This is consistent with the IPQ results, which revealed a very realistic representation in the VE of the real experience using the EPW for both tests using an HMD with a joystick, and a projector together with the eye tracker. Furthermore, these results show clearly that using an HMD increases the sense of presence, making the experience more immersive and realistic.

Considering the overall results, it is concluded here that for this stage of our research, the Simcadrom simulates a real EPW close enough. So, we believe it can be used for virtual training using a joystick and an HMD, or an eye tracker as an alternative interface and a projector.

**Hypothesis** **2.** *Improvement During Training*.

There is a clear relationship between the time parameter and the path following error because of the way the test protocol was designed. For most participants, reducing their total elapsed times during the training was more important than reducing their path following error. The subjects received feedback during training on their elapsed times from the simulator and not on their number of commands or their path following error, other than having to pass over the letters marked on the floor. Also, in the RE they could see an examiner taking time with a stopwatch. Therefore, the path following error ended up not being a good comparison parameter for determining performance improvement, at least not in these tests’ conditions. Overall, there was no significant difference (*p* > 0.05) between the mean values per trials in the VE or RE using a joystick nor eye tracker (see Section 4.3 and Section 4.4). Additionally, seemed like six trials were not enough to notice improvements or even just changes for all comparison parameters in every condition.

However, from “comparison 2”, we can see that when subjects trained using the joystick and an HMD in the VE and the RE, they improved their total elapsed times completing the tasks, reduced their number of commands in the RE training, and the number of commands of the group training in the VE became more homogeneous. Also, participants reduced the total number of commands they made during the virtual training using the eye tracker and reduced the variability when training in the RE.

When training in the RE with the eye tracker, subjects improved their elapsed time. Also, it is worth mentioning that their results from the path following error and number of commands in the RE became more homogeneous. From “Comparison 4” we can also evidence improvement of the elapsed time in the opposite environment when training in the VE with the joystick. The same happened for the group that trained in the RE using the eye tracker, they improved their elapsed time and their number of commands in the opposite environment. Consequently, participants were able to learn and improve their EPW driving skills by doing a training session in the Simcadrom.

Still, half of the participants that used the HMD indicated they felt nausea, whereas 90% of the participants that used the projector did not experience it. This suggests that the tests with HMD caused the participants to experience cybersickness. Although not everyone that used the HMD felt dizzy, this could represent a big setback for long training sessions.

**Hypothesis** **3.** *Skills Transferred to the Opposite Environment*.

The quantitative data that aids in the discussion about the hypothesis 3, comes from “Comparison 3” that tries to measure if the performance is maintained in the opposite environment, “Comparison 4” that aims to measure if the skills improvement continued or was at least maintained in the opposite environment, and “Comparison 5”, which measures if there was evidence of improving skills after a training session in the opposite environment.

When subjects trained with the joystick and the HMD in the VE and RE, their performance in the opposite environment to where they trained seemed to not be better than the performance of those who didn’t trained (”Comparison 5”), e.g., “RJ VT” was not better than “RJ T1”.

However, from “Comparison 4” we can see that the last obtained time in the RE (“RJ VT”) was significantly smaller than the values from the first trial in the VE (“VJ T1”), suggesting that the learning process continued even in the RE. Also, the performance in time and number of commands for the VE was considered to be maintained after training in the RE with the joystick.

Additionally, “Comparison 3” also suggests that the participants’ performance measured in time in the RE was maintained when using the virtual EPW after the training in the RE. The same was evidenced for the path following error (in both RE and VE) and for the number of commands when the training session was in the VE.

It was noticed that completing the task in the RE felt easier by the participants using the joystick than using the eye tracker interface. Driving the EPW with the eye tracker is indeed more difficult, however, the participants who used it reported using previously the virtual or real EPW helped them more to complete the task in the opposite environment than the group of participants that used the joystick.

From “Comparison 3” and “Comparison 4” we can also evidence that the mean time from the first trial in the VE with no previous training, was significantly greater than the time from the first trial in the VE after training. This indicates that the training in the RE with eye tracker helped participants to perform better in the VE than those without training. The performance in the path following error was also maintained in RE after training in the VE. The number of commands using the eye tracker was also maintained in the opposite environment.

Therefore, we believe the skills learned in the training session in the Simcadrom can be transferred to the real EPW. Yet some enhancements in the test protocol are required to evidence more improvements. It was aforementioned that although six trials can be considered an acceptable parameter for the test protocol, it may not be enough trials to see improvements in all parameters, and even if they are, they may not be enough to notice a skill transfer to the opposite environment.

## 6. Future Work

Next test protocol designs for this Simulator will need to evaluate, based on this experience, the number of trials and the time between each trial considering all the logistic and practical implications. Another important factor to consider in future work is the need of reducing cybersickness considering vestibular feedback, particularly if using an HMD.

Although there is room for improvement in the EPW behavior modeling and its VE, we are satisfied with the realism and immersion results of the selected VE. Then, future work may not have the need to focus on recreating an existing physical environment for comparison purposes and focus more on building virtual scenarios aligned with established wheelchair assessment and training protocols to address realistic needs for end-user groups with motor disabilities.

## Figures and Tables

**Table 1:** Elapsed time statistical test for virtual and real training with a joystick (“Comparison 1”).

**SW**	0.976	0.868	0.837	0.761	0.689	0.914
**Critical Wα**	0.806	0.806	0.806	0.762	0.686	0.806
***p*-Value**	0.915	0.258	0.155	0.038	0.012	0.490
**F**	3.852	1.556	4.538	1.060	1.458	1.614
**Num df**	4	4	3	4	4	4
**Den df**	4	4	4	4	4	4
***p*-Value**	0.110	0.339	0.089	0.478	0.362	0.327
**t**	1.365	0.043	0.213	0.227	0.131	0.970
**df**	8	8	7	8	8	8
***p*-Value**	0.209	0.967	0.837	0.826	0.899	0.361

**Table 2:** T-test of mean elapsed time. Comparison 2, 3, 4, and 5 from the VE and RE using a joystick.

Comparison				Mean (s)	Variance (s^2^)	*t*-Test Type	*t*	Critical *t*	df	*p*-Value
#		Between								
	2		VJ T1	49.156	276.458	Paired one-tailed	3.166	2.132	4	**0.017**
			VJ T6	30.916	22.717					
	3		RJ VT	37.344	35.200	Paired two-tailed	4.214	2.776	4	**0.014**
			VJ T6	30.916	22.717					
	4		VJ T1	49.156	276.458	Paired one-tailed	2.215	2.132	4	**0.046**
			RJ VT	37.344	35.200					
	5		RJ T1	37.766	71.764	Unpaired one-tailed equal variances	0.091	1.860	8	**0.465**
			RJ VT	37.344	35.200					
	2		RJ T1	37.766	71.764	Paired one-tailed	2.656	2.132	4	**0.028**
			RJ T6	28.286	14.071					
	3		VJ RT	45.952	353.539	Paired two-tailed	2.460	2.776	4	**0.070**
			RJ T6	28.286	14.071					
	4		VJ RT	45.952	353.539	Paired one-tailed	0.969	2.132	4	**0.194**
			RJ T1	37.766	71.764					
	5		VJ T1	49.156	276.458	Unpaired one-tailed unequal variances	0.285	1.860	8	**0.391**
			VJ RT	45.952	353.539					

## Supplementary Materials

The supplementary materials are available online at https://www.mdpi.com/1424-8220/20/12/3565/s1.

## References

[1] United Nations Department of Economic and Social Affairs (2019). Disability and Development Report: Realizing the Sustainable Development Goals by, for and with Persons with Disabilities.

[2] Fehr L., Langbein W.E., Skaar S.B. (2000). Adequacy of power wheelchair control interfaces for persons with severe disabilities: A clinical survey. J. Rehabil. Res. Dev..

[3] Bastos-Filho T.F., Kumar D.K., Arjunan S.P. (2014). Devices for Mobility and Manipulation for People with Reduced Abilities (Rehabilitation Science in Practice Series).

[4] Dicianno B.E., Sibenaller S., Kimmich C., Cooper R.A., Pyo J. (2009). Joystick use for virtual power wheelchair driving in individuals with tremor: Pilot study. J. Rehabil. Res. Dev..

[5] Huang D., Qian K., Fei D.Y., Jia W., Chen X., Bai O. (2012). Electroencephalography (EEG)-based brain-computer interface (BCI): A 2-D virtual wheelchair control based on event-related desynchronization/synchronization and state control. IEEE Trans. Neural Syst. Rehabil. Eng..

[6] Kaiser M.S., Iqbal Z., Shamim C., Mamun A., Mahmud H.M. (2016). A Neuro-Fuzzy Control System Based on Feature Extraction of Surface Electromyogram Signal for Solar Powered Wheelchair. Cogn. Comput..

[7] Purwanto D., Mardiyanto R., Arai K. (2009). Electric wheelchair control with gaze direction and eye blinking. Artif. Life Robot..

[8] Leeb R., Sagha H., Chavarriaga R., Millán J.D.R. (2011). A hybrid brain-computer interface based on the fusion of electroencephalographic and electromyographic activities. J. Neural Eng..

[9] Martins F.N., Celeste W.C., Carelli R., Sarcinelli-Filho M., Bastos-Filho T.F. (2008). An adaptive dynamic controller for autonomous mobile robot trajectory tracking. Control Eng. Pract..

[10] Viswanathan P., Wang R.H., Sutcliffe A., Kenyon L., Foley G., Miller W.C., Bell J., Kirby L., Simpson R., Mihailidis A. (2018). Smart Wheelchairs in Assessment and Training ( SWAT ): State of the Field. In Proceedings of the Age-Well NCE Position Paper. https://agewell-nce.ca/.

[11] Borges L.R., Martins F.R., Naves E.L.M., Bastos T.F., Lucena V.F. (2016). Multimodal System for Training at Distance in a Virtual or Augmented Reality Environment for Users of Electric-Powered Wheelchairs. IFAC PapersOnLine.

[12] Song Z., Guo S., Yazid M. Development of a potential system for upper limb rehabilitation training based on virtual reality. Proceedings of the 4th International Conference on Human System Interaction (HSI).

[13] Pithon T., Weiss T., Richir S., Klinger E. (2009). Wheelchair simulators: A review. Technol. Disabil..

[14] Faria B.M., Reis L.P., Lau N. (2014). A survey on intelligent wheelchair prototypes and simulators. Advances in Intelligent Systems and Computing.

[15] Tao G., Archambault P.S. (2016). Powered wheelchair simulator development: Implementing combined navigation-reaching tasks with a 3D hand motion controller. J. Neuroeng. Rehabil..

[16] Adelola I.A., Cox S.L., Rahman A. (2009). Virtual environments for powered wheelchair learner drivers: Case studies. Technol. Disabil..

[17] Braga R.A.M., Petry M., Moreira A.P., Reis L.P. Intellwheels—A development platform for intelligent wheelchairs for disabled people. Proceedings of the 5th International Conference on Informatics in Control, Automation and Robotics (ICINCO 2008).

[18] Headleand C.J., Day T., Pop S.R., Ritsos P.D., John N.W. (2016). A Cost-Effective Virtual Environment for Simulating and Training Powered Wheelchairs Manoeuvres. Stud. Health Technol. Inform..

[19] Day T.W., Dobson W.H., Headleand C.J., John N.W., Pop S.R. Using virtual reality to experience different powered wheelchair configurations. Proceedings of the 2017 International Conference on Cyberworlds (CW 2017) in cooperation with: Eurographics Association International Federation for Information Processing (ACM SIGGRAPH).

[20] John N.W., Pop S.R., Day T.W., Ritsos P.D., Headleand C.J. (2018). The Implementation and Validation of a Virtual Environment for Training Powered Wheelchair Manoeuvres. IEEE Trans. Vis. Comput. Graph..

[21] Archambault P.S., Tremblay S., Cachecho S., Routhier F., Boissy P. (2012). Driving performance in a power wheelchair simulator. Disabil. Rehabil. Assist. Technol..

[22] Archambault P.S., Chong J.N.F., Sorrento G., Routhier F., Boissy P. Comparison of powered wheelchair driving performance in a real and in a simulated environment. Proceedings of the International Conference on Virtual Rehabilitation 2011.

[23] Vailland G., Gaffary Y., Devigne L., Gouranton V., Arnaldi B., Babel M. Vestibular feedback on a virtual reality wheelchair driving simulator: A pilot study. Proceedings of the ACM/IEEE International Conference on Human-Robot Interaction.

[24] Montenegro-Couto E.H., Hernandez-Ossa K.A., Bissoli A.L.C., Sime M., Bastos-Filho T.F. Towards an assistive interface to command robotic wheelchairs and interact with environment through eye gaze. Proceedings of the V Congresso Brasileiro de Eletromiografia e Cinesiologia e X Simpósio de Engenharia Biomédica, Even3.

[25] Fuchs P., Hafez M., Benali Koudja M., Papin J.-P., Tsingos N., Warusfel O. (2006). Les sens de l’homme. Traité de la Réalité Virtuelle.

[26] Adelola I.A., Cox S.L., Rahman A. Adaptable virtual reality interface for powered wheelchair training of disabled children. Proceedings of the 4th International Conference on Disability, Virtual Reality and Associated Technologies.

[27] Inman D.P., Loge K., Cram A., Peterson M. (2011). Learning to Drive a Wheelchair in Virtual Reality. J. Spec. Educ. Technol..

[28] Mahajan H.P., Dicianno B.E., Cooper R.A., Ding D. (2013). Assessment of wheelchair driving performance in a virtual reality-based simulator. J. Spinal Cord Med..

[29] Rebenitsch L., Owen C. (2016). Review on cybersickness in applications and visual displays. Virtual Real..

[30] Harrison A., Derwent G., Enticknap A., Rose F.D., Attree E.A. (2002). The role of virtual reality technology in the assessment and training of inexperienced powered wheelchair users. Disabil. Rehabil..

[31] Rivera H., Hernandez-Ossa K.A., Longo B., Caldeira E., Bastos T. Evaluation of Cybersickness and Sense of Presence in a VR Simulator of Electric-Powered Wheelchairs. Proceedings of the 2nd International Workshop on Assistive Technology.

[32] Sonar A.V., Burdick K.D., Begin R.R., Resch E.M., Thompson E.M., Thacher E., Searleman J., Fulk G., Carroll J.J. Development of a Virtual Reality-based power wheel chair simulator. Proceedings of the IEEE International Conference on Mechatronics and Automation, ICMA 2005.

[33] Hasdai A., Jessel A.S., Weiss P.L. (1998). Use of a Computer Simulator for Training Children with Disabilities in the Operation of a Powered Wheelchair. Am. J. Occup. Ther..

[34] Spaeth D.M., Mahajan H., Karmarkar A., Collins D., Cooper R.A., Boninger M.L. (2008). Development of a Wheelchair Virtual Driving Environment: Trials With Subjects With Traumatic Brain Injury. Arch. Phys. Med. Rehabil..

[35] Schubert T., Friedmann F., Regenbrecht H. (2001). The Experience of Presence: Factor Analytic Insights. Presence Teleoperators Virtual Environ..

[36] Ganier F., Hoareau C., Tisseau J. (2014). Evaluation of procedural learning transfer from a virtual environment to a real situation: A case study on tank maintenance training. Ergonomics.

[37] Kozak J.J., Hancock P.A., Arthur E.J., Chrysler S.T. (1993). Transfer of training from virtual reality. Ergonomics.

[38] Seymour N.E., Gallagher A.G., Roman S.A., O’Brien M.K., Bansal V.K., Andersen D.K., Satava R.M. (2002). Virtual Reality Training Improves Operating Room Performance. Ann. Surg..

[39] Yao R., Heath T., Davies A., Forsyth T., Mitchell N., Hoberman P. (2014). Oculus VR Best Practices Guide. Oculus VR.

[40] Miranda Lessa H.C., Bastos-Filho T.F., Frizera-Neto A. Localização de Cadeiras de Rodas Baseada na Fusão de Sinais de Sensor Inercial e Odometria, Federal University of Espirito Santo, Brazil, 2017. https://www.scribd.com/document/464367342/LOCALIZACAO-DE-CADEIRA-DE-RODAS-BASEADA-NA-FUSAO-DE-SINAIS-DE-SENSORES-INERCIAIS-E-ODOMETRIA.

[41] Dawson D.R., Kaiserman-Goldenstein E., Chan R., Gleason J. (2006). Power-Mobility Indoor Driving Assessment Manual (PIDA).

[42] Kirby R.L. (2016). Wheelchair Skills Assessment and Training.

[43] Mahajan H., Spaeth D.M., Dicianno B.E., Collins D.M., Boninger M.L., Cooper R.A. (2012). Comparison of virtual wheelchair driving performance of people with TBI using an isometric and a conventional joystick. Arch. Phys. Med. Rehabil..

[44] Ganier F., Hoareau C., Devillers F. (2013). Learning a Procedural Task in a Virtual Environment for Training: Evaluation of Performance and Workload. Trav. Hum..

[45] Vasconcelos-Raposo J., Bessa M., Melo M., Barbosa L., Rodrigues R., Teixeira C.M., Cabral L., Sousa A.A. (2016). Adaptation and Validation of the Igroup Presence Questionnaire (IPQ) in a Portuguese Sample. Presence Teleoperators Virtual Environ..

[46] Armstrong R.A. (2014). When to use the Bonferroni correction. Ophthalmic Physiol. Opt..

[47] Gignac G.E. [how2stats] Is the Bonferroni Correction Really Necessary?. https://youtu.be/kpTvTyMcDqY.

[48] Ahad N.A., Teh S.Y., Othiman A.R., Yaacob C.R. (2011). Sensitivity of Normality Tests to Non-normal Data. Sains Malays..

[49] Robbins N.B., Heiberger R.M. Plotting Likert and Other Rating Scales. Proceedings of the Joint Statistical Meetings 2011.

