# Supplementary material for: Simulation System of Electric-Powered Wheelchairs for Training Purposes
Source: Sensors (Basel). 2020 Jun 24;20(12):3565. doi: 10.3390/s20123565 (PMC7348882; doi:10.3390/s20123565)
Supplement: Supplementary file 1 [file sensors-20-03565-s001.zip › sensors-786419-supplementary.docx]

Supplementary Materials for the Work Titled “Simulation System of Electric-Powered Wheelchairs for Training Purposes”

A. Elapsed Times for Virtual and Real Training Using a Joystick.

**Table S1.** Elapsed times for virtual training using a joystick.

| **Participant** |  | **VJ Elapsed Times [s] Per Trial During the Training** | | | | |  | **RJ VT [s]** |
| --- | --- | --- | --- | --- | --- | --- | --- | --- |
|  | **1** | **2** | **3** | **4** | **5** | **6** | **7** | |
| 1 | 35.22 | 27.63 | 25.26 | 27.47 | 26.32 | 25.24 | 29.09 | |
| 2 | 55.06 | 36.66 | 35.27 | 36.96 | 30.73 | 33.80 | 37.03 | |
| 3 | 33.18 | 26.06 | 27.36 | 25.77 | 26.84 | 26.21 | 35.77 | |
| 4 | 48.25 | 33.91 | - | 39.08 | 36.06 | 34.47 | 39.36 | |
| 5 | 74.07 | 42.91 | 50.23 | 35.65 | 32.13 | 34.86 | 45.47 | |
| **Mean** | **49.16** | **33.43** | **34.53** | **32.99** | **30.42** | **30.92** | **37.34** | |
| **SD** | **14.87** | **6.14** | **9.80** | **5.34** | **3.59** | **4.26** | **5.31** | |

**Table S2.** Elapsed times for real training using a joystick.

| **Participant** |  | **RJ Elapsed Times [s] Per Trial During the Training** | | | | |  | **VJ RT [s]** |
| --- | --- | --- | --- | --- | --- | --- | --- | --- |
|  | **1** | **2** | **3** | **4** | **5** | **6** | **7** | |
| 6 | 25.23 | 23.96 | 24.99 | 23.97 | 23.08 | 23.02 | 30.27 | |
| 7 | 35.89 | 35.11 | 35.20 | 30.37 | 28.37 | 30.99 | 41.27 | |
| 8 | 36.41 | 38.42 | 35.03 | 39.93 | 36.25 | 32.37 | 71.84 | |
| 9 | 45.31 | 33.44 | 39.29 | 32.31 | 32.04 | 26.22 | 28.01 | |
| 10 | 45.99 | 35.40 | 32.12 | 34.12 | 30.50 | 28.83 | 58.37 | |
| **Mean** | **37.77** | **33.27** | **33.33** | **32.14** | **30.05** | **28.29** | **45.95** | |
| **SD** | **7.58** | **4.92** | **4.75** | **5.19** | **4.34** | **3.36** | **16.82** | |

**Table S3.** Elapsed time statistical test for virtual and real training with a joystick (Comparison 1).

| **Trial** | **1** | **2** | **3** | **4** | **5** | **6** |
| --- | --- | --- | --- | --- | --- | --- |
| SW | 0.976 | 0.868 | 0.837 | 0.761 | 0.689 | 0.914 |
| Critical Wα | 0.806 | 0.806 | 0.806 | 0.762 | 0.686 | 0.806 |
| **p-value** | **0.915** | **0.258** | **0.155** | **0.038** | **0.012** | **0.490** |
| F | 3.852 | 1.556 | 4.538 | 1.060 | 1.458 | 1.614 |
| Num df | 4 | 4 | 3 | 4 | 4 | 4 |
| Den df | 4 | 4 | 4 | 4 | 4 | 4 |
| **p-value** | **0.110** | **0.339** | **0.089** | **0.478** | **0.362** | **0.327** |
| t | 1.365 | 0.043 | 0.213 | 0.227 | 0.131 | 0.970 |
| df | 8 | 8 | 7 | 8 | 8 | 8 |
| **p-value** | **0.209** | **0.967** | **0.837** | **0.826** | **0.899** | **0.361** |

**Table S4.** T-test of mean elapsed time. Comparison 2, 3, 4 and 5 from the VE and RE using a joystick.

| **Comparison** | | | | **Mean**  **[s]** | **Variance**  **[s^2^]** | **t-test Type** | **t** | **Critical**  **t** | **df** | **p-Value** |
| --- | --- | --- | --- | --- | --- | --- | --- | --- | --- | --- |
| # | | **Between** | |  |  |  |  |  |  |  |
|  | 2 |  | VJ T1 | 49.156 | 276.458 | Paired one-tailed | 3.166 | 2.132 | 4 | **0.017** |
|  |  |  | VJ T6 | 30.916 | 22.717 |  |  |  |  |  |
|  | 3 |  | RJ VT | 37.344 | 35.200 | Paired two-tailed | 4.214 | 2.776 | 4 | **0.014** |
|  |  |  | VJ T6 | 30.916 | 22.717 |  |  |  |  |  |
|  | 4 |  | VJ T1 | 49.156 | 276.458 | Paired one-tailed | 2.215 | 2.132 | 4 | **0.046** |
|  |  |  | RJ VT | 37.344 | 35.200 |  |  |  |  |  |
|  | 5 |  | RJ T1 | 37.766 | 71.764 | Unpaired one-tailed equal variances | 0.091 | 1.860 | 8 | **0.465** |
|  |  |  | RJ VT | 37.344 | 35.200 |  |  |  |  |  |
|  | 2 |  | RJ T1 | 37.766 | 71.764 | Paired one-tailed | 2.656 | 2.132 | 4 | **0.028** |
|  |  |  | RJ T6 | 28.286 | 14.071 |  |  |  |  |  |
|  | 3 |  | VJ RT | 45.952 | 353.539 | Paired two-tailed | 2.460 | 2.776 | 4 | **0.070** |
|  |  |  | RJ T6 | 28.286 | 14.071 |  |  |  |  |  |
|  | 4 |  | VJ RT | 45.952 | 353.539 | Paired one-tailed | 0.969 | 2.132 | 4 | **0.194** |
|  |  |  | RJ T1 | 37.766 | 71.764 |  |  |  |  |  |
|  | 5 |  | VJ T1 | 49.156 | 276.458 | Unpaired one-tailed unequal variances | 0.285 | 1.860 | 8 | **0.391** |
|  |  |  | VJ RT | 45.952 | 353.539 |  |  |  |  |  |

B. Elapsed Times for Virtual and Real Training Using the Eye Tracker.

**Table S5.** Elapsed times for virtual training using the eye tracker.

| **Participant** |  | **VET Elapsed Times [s] Per Trial During the Training** | | | | |  | **RET VT [s]** |
| --- | --- | --- | --- | --- | --- | --- | --- | --- |
|  | **1** | **2** | **3** | **4** | **5** | **6** | **7** | |
| 11 | 178.83 | 253.35 | 158.70 | 190.19 | 155.39 | 144.37 | 286.26 | |
| 12 | 135.92 | 147.57 | 137.27 | 129.68 | 131.39 | 161.84 | 212.19 | |
| 13 | 259.59 | 180.30 | 233.96 | 205.20 | 274.86 | 262.03 | 372.54 | |
| 14 | 190.32 | 187.21 | 143.06 | 148.71 | 136.62 | 145.16 | 200.09 | |
| 15 | - | 139.85 | - | 123.13 | 118.71 | - | 253.63 | |
| **Mean** | **191.17** | **181.66** | **168.25** | **159.38** | **163.39** | **178.35** | **264.94** | |
| **SD** | **44.40** | **40.21** | **38.74** | **32.74** | **56.97** | **48.81** | **61.88** | |

**Table S6.** Elapsed times for real training using the eye tracker.

| **Participant** |  | **RET Elapsed Times [s] Per Trial During the Training** | | | | |  | **VET RT [s]** |
| --- | --- | --- | --- | --- | --- | --- | --- | --- |
|  | **1** | **2** | **3** | **4** | **5** | **6** | **7** | |
| 16 | 399.74 | 242.89 | 267.16 | 199.84 | 272.68 | 193.33 | 129.82 | |
| 17 | 396.98 | 218.06 | 196.41 | 241.51 | 223.06 | 259.70 | 174.67 | |
| 18 | 296.55 | 226.83 | 238.26 | 183.86 | 246.33 | 225.16 | 126.62 | |
| 19 | 217.89 | 230.09 | 224.05 | 228.10 | 200.36 | 236.11 | 142.83 | |
| 20 | 336.10 | 236.30 | 254.05 | 236.94 | 201.11 | 286.44 | 132.64 | |
| **Mean** | **329.45** | **230.83** | **235.99** | **218.05** | **228.71** | **240.15** | **141.32** | |
| **SD** | **67.93** | **8.43** | **24.54** | **22.40** | **27.72** | **31.49** | **17.54** | |

**Table S7.** Elapsed time statistical test for virtual and real training with eye tracker. (Comparison 1).

| **Trial** | **1** | **2** | **3** | **4** | **5** | **6** |
| --- | --- | --- | --- | --- | --- | --- |
| SW | 0.822 | 0.960 | 0.905 | 0.868 | 0.860 | 0.861 |
| Critical Wα | 0.806 | 0.806 | 0.806 | 0.806 | 0.806 | 0.806 |
| **p-value** | **0.148** | **0.808** | **0.436** | **0.259** | **0.228** | **0.264** |
| F | 2.194 | 22.768 | 2.657 | 2.136 | 4.224 | 2.563 |
| Num df | 4 | 4 | 3 | 4 | 4 | 3 |
| Den df | 3 | 4 | 4 | 4 | 4 | 4 |
| **p-value** | **0.272** | **0.005** | **0.184** | **0.240** | **0.096** | **0.193** |
| t | 3.100 | 2.394 | 2.814 | 2.958 | 2.062 | 2.025 |
| df | 7 | 4 | 7 | 8 | 8 | 7 |
| **p-value** | **0.017** | **0.075** | **0.026** | **0.018** | **0.073** | **0.083** |

**Table S8.** T-test of mean elapsed time. Comparison 2, 3, 4, and 5 from the VE and RE using eye tracker.

| **Comparison** | | | | **Mean**  **[s]** | **Variance**  **[s^2^]** | **t-test**  **Type** | **t** | **Critical t** | **df** | **p-Value** |
| --- | --- | --- | --- | --- | --- | --- | --- | --- | --- | --- |
| **#** | | **Between** | |  |  |  |  |  |  |  |
|  | 2 |  | VET T1 | 191.165 | 2628.952 | Paired  one-tailed | 0.779 | 2.353 | 3 | 0.246 |
|  |  |  | VET T6 | 178.35 | 3177.046 |  |  |  |  |  |
|  | 3 |  | RET VT | 267.77 | 6329.451 | Paired  two-tailed | 4.028 | 3.182 | 3 | 0.028 |
|  |  |  | VET T6 | 178.35 | 3177.046 |  |  |  |  |  |
|  | 4 |  | RET VT | 267.77 | 6329.451 | Paired  one-tailed | 3.233 | 2.353 | 3 | 0.024 |
|  |  |  | VET T1 | 191.165 | 2628.952 |  |  |  |  |  |
|  | 5 |  | RET T1 | 329.452 | 5768.313 | Unpaired  one-tailed  equal  variances | 1.404 | 1.860 | 8 | 0.099 |
|  |  |  | RET VT | 264.942 | 4787.076 |  |  |  |  |  |
|  | 2 |  | RET T1 | 329.452 | 5768.313 | Paired  one-tailed | 2.326 | 2.132 | 4 | 0.040 |
|  |  |  | RET T6 | 240.148 | 1239.525 |  |  |  |  |  |
|  | 3 |  | RET T6 | 240.148 | 1239.525 | Paired  two-tailed | 6.596 | 2.776 | 4 | 0.003 |
|  |  |  | VET RT | 141.316 | 384.5462 |  |  |  |  |  |
|  | 4 |  | RET T1 | 329.452 | 5768.313 | Paired  one-tailed | 5.777 | 2.132 | 4 | 0.002 |
|  |  |  | VET RT | 141.316 | 384.5462 |  |  |  |  |  |
|  | 5 |  | VET T1 | 191.165 | 2628.952 | Unpaired  one-tailed  equal  variances | 2.025 | 1.895 | 7 | 0.041 |
|  |  |  | VET RT | 141.316 | 384.5462 |  |  |  |  |  |

C. Path Following Error in Virtual and Real training Using a Joystick.

**Table S9.** Path following error for virtual training using a joystick.

| **Participant** |  | **VJ RMSE [m] Per Trial During the Training** | | | | |  | **RJ VT [m]** |
| --- | --- | --- | --- | --- | --- | --- | --- | --- |
|  | **1** | **2** | **3** | **4** | **5** | **6** | **7** | |
| 1 | 0.2338 | 0.1816 | 0.2030 | 0.1262 | 0.1627 | 0.1333 | 0.2489 | |
| 2 | 0.1456 | 0.1214 | 0.1975 | 0.1695 | 0.1650 | 0.2003 | 0.1951 | |
| 3 | 0.2352 | 0.1705 | 0.1584 | 0.1185 | 0.1212 | 0.1328 | 0.1339 | |
| 4 | 0.2617 | 0.3128 | 0.3798 | 0.1543 | 0.1831 | 0.2444 | 0.2920 | |
| 5 | 0.1011 | 0.1406 | 0.1783 | 0.1757 | 0.1724 | 0.1690 | 0.2175 | |
| **Mean** | **0.1955** | **0.1854** | **0.2234** | **0.1488** | **0.1609** | **0.1760** | **0.2175** | |
| **SD** | **0.0613** | **0.0672** | **0.0798** | **0.0228** | **0.0211** | **0.0424** | **0.0530** | |

**Table S10.** Path following error for real training using a joystick.

| **Participant** |  | **RJ RMSE [m] Per Trial During the Training** | | | | |  | **VJ RT [m]** |
| --- | --- | --- | --- | --- | --- | --- | --- | --- |
|  | **1** | **2** | **3** | **4** | **5** | **6** | **7** | |
| 6 | 0.1587 | 0.1477 | 0.1215 | 0.1326 | 0.1869 | 0.2090 | 0.2321 | |
| 7 | 0.2150 | 0.1502 | 0.1244 | 0.1401 | - | - | 0.1760 | |
| 8 | 0.1766 | 0.2619 | 0.2709 | 0.3504 | 0.2312 | 0.3992 | 0.1992 | |
| 9 | 0.3252 | 0.3034 | 0.1670 | 0.2257 | 0.2077 | 0.2085 | 0.3012 | |
| 10 | 0.1720 | 0.1487 | 0.1499 | 0.1727 | 0.2027 | 0.2697 | 0.2138 | |
| **Mean** | **0.2095** | **0.2024** | **0.1667** | **0.2043** | **0.2071** | **0.2716** | **0.2244** | |
| **SD** | **0.0608** | **0.0668** | **0.0547** | **0.0801** | **0.0159** | **0.0778** | **0.0425** | |

**Table S11.** Path following error statistical test for virtual and real training with a joystick (Comparison 1).

| **Trial** | **1** | **2** | **3** | **4** | **5** | **6** |
| --- | --- | --- | --- | --- | --- | --- |
| SW | 0.745 | 0.947 | 0.854 | 0.827 | 0.822 | 0.984 |
| Critical Wα | 0.715 | 0.806 | 0.806 | 0.806 | 0.806 | 0.806 |
| **p-value** | **0.027** | **0.713** | **0.207** | **0.132** | **0.120** | **0.953** |
| F | 1.018 | 1.010 | 2.125 | 12.285 | 1.648 | 3.581 |
| N df | 4 | 4 | 4 | 4 | 4 | 3 |
| D df | 4 | 4 | 4 | 4 | 3 | 4 |
| **p-value** | **0.493** | **0.496** | **0.242** | **0.016** | **0.355** | **0.125** |
| t | 0.325 | 0.359 | 1.172 | 1.332 | 3.213 | 2.070 |
| df | 8 | 8 | 8 | 5 | 7 | 7 |
| **p-value** | **0.754** | **0.729** | **0.275** | **0.240** | **0.015** | **0.077** |

**Table S12.** T-test of path following errors. Comparison 2, 3, 4, and 5 from the VE and RE using a joystick.

| **Comparison** | | | | **Mean**  **[m]** | **Variance**  **[m^2^]** | **t-test Type** | **t** | **Critical t** | **df** | **p-Value** |
| --- | --- | --- | --- | --- | --- | --- | --- | --- | --- | --- |
| **#** | | **Between** | |  |  |  |  |  |  |  |
|  | 2 |  | VJ T1 | 0.195 | 0.005 | Paired one-tailed | 0.536 | 2.132 | 4 | 0.310 |
|  |  |  | VJ T6 | 0.176 | 0.002 |  |  |  |  |  |
|  | 3 |  | RJ VT | 0.217 | 0.004 | Paired two-tailed | 1.917 | 2.776 | 4 | 0.128 |
|  |  |  | VJ T6 | 0.176 | 0.002 |  |  |  |  |  |
|  | 4 |  | RJ VT | 0.217 | 0.004 | Paired one-tailed | 0.622 | 2.132 | 4 | 0.284 |
|  |  |  | VJ T1 | 0.195 | 0.005 |  |  |  |  |  |
|  | 5 |  | RJ VT | 0.217 | 0.004 | Unpaired one-tailed equal variances | 0.198 | 1.860 | 8 | 0.424 |
|  |  |  | RJ T1 | 0.210 | 0.005 |  |  |  |  |  |
|  | 2 |  | RJ T6 | 0.272 | 0.008 | Paired one-tailed | 0.904 | 2.353 | 3 | 0.216 |
|  |  |  | RJ T1 | 0.208 | 0.006 |  |  |  |  |  |
|  | 3 |  | RJ T6 | 0.272 | 0.008 | Paired two-tailed | 0.558 | 3.182 | 3 | 0.616 |
|  |  |  | VJ RT | 0.237 | 0.002 |  |  |  |  |  |
|  | 4 |  | VJ RT | 0.224 | 0.002 | Paired one-tailed | 0.719 | 2.132 | 4 | 0.256 |
|  |  |  | RJ T1 | 0.210 | 0.005 |  |  |  |  |  |
|  | 5 |  | VJ RT | 0.224 | 0.002 | Unpaired one-tailed equal variances | 0.776 | 1.860 | 8 | 0.230 |
|  |  |  | VJ T1 | 0.195 | 0.005 |  |  |  |  |  |

D. Path Following Error in Virtual and Real Training Using Eye Tracker.

**Table S13.** Path following error for virtual training using eye tracker.

| **Participant** |  | **VET RMSE [m] Per Trial During the Training** | | | | |  | **RET VT [m]** |
| --- | --- | --- | --- | --- | --- | --- | --- | --- |
|  | **1** | **2** | **3** | **4** | **5** | **6** | **7** | |
| 11 | 0.30 | 0.38 | 0.34 | 0.40 | 0.33 | 0.33 | 0.2804 | |
| 12 | 0.21 | 0.25 | 0.34 | 0.28 | 0.32 | 0.33 | 0.3193 | |
| 13 | 0.37 | 0.35 | 0.46 | 0.50 | 0.44 | 0.37 | 0.5243 | |
| 14 | 0.24 | 0.24 | 0.17 | 0.26 | 0.27 | 0.32 | 0.3947 | |
| 15 | 0.36 | 0.38 | 0.27 | 0.30 | - | - | 0.2335 | |
| **Mean** | **0.2957** | **0.3212** | **0.3170** | **0.3474** | **0.3406** | **0.3388** | **0.3504** | |
| **SD** | **0.0662** | **0.0616** | **0.0954** | **0.0895** | **0.0637** | **0.0199** | **0.1017** | |

**Table S14.** Path following error for real training using eye tracker.

| **Participant** |  | **RET RMSE [m] Per Trial During the Training** | | | | |  | **VET RT [m]** |
| --- | --- | --- | --- | --- | --- | --- | --- | --- |
|  | **1** | **2** | **3** | **4** | **5** | **6** | **7** | |
| 16 | 0.21 | 0.38 | 0.52 | 0.51 | 0.62 | 0.43 | 0.3621 | |
| 17 | 0.34 | 0.68 | 0.57 | 0.66 | 0.69 | 0.47 | 0.3714 | |
| 18 | 0.30 | 0.34 | 0.25 | 0.52 | 0.33 | - | 0.3259 | |
| 19 | 0.25 | 0.42 | 0.52 | 0.37 | 0.37 | 0.41 | 0.4498 | |
| 20 | 0.22 | 0.38 | 0.41 | 0.40 | 0.56 | 0.46 | 0.2496 | |
| **Mean** | **0.2642** | **0.4395** | **0.4544** | **0.4926** | **0.5147** | **0.4438** | **0.3518** | |
| **SD** | **0.0506** | **0.1221** | **0.1151** | **0.1012** | **0.1401** | **0.0265** | **0.0651** | |

**Table S15.** Path following error statistical test for virtual and real training with eye tracker (Comparison 1).

| **Trial** | **1** | **2** | **3** | **4** | **5** | **6** |
| --- | --- | --- | --- | --- | --- | --- |
| SW | 0.940 | 0.768 | 0.890 | 0.776 | 0.956 | 0.826 |
| Critical Wα | 0.806 | 0.762 | 0.806 | 0.762 | 0.806 | 0.806 |
| **p-value** | **0.665** | **0.043** | **0.356** | **0.051** | **0.753** | **0.179** |
| F | 1.707 | 3.936 | 1.454 | 1.279 | 4.534 | 1.773 |
| N df | 4 | 4 | 4 | 4 | 4 | 3 |
| D df | 4 | 4 | 4 | 4 | 3 | 3 |
| **p-value** | **0.309** | **0.107** | **0.363** | **0.409** | **0.122** | **0.325** |
| t | 0.756 | 1.730 | 1.837 | 2.148 | 2.030 | 5.491 |
| df | 8 | 8 | 8 | 8 | 7 | 6 |
| **p-value** | **0.472** | **0.122** | **0.103** | **0.064** | **0.082** | **0.002** |

**Table S16.** T-test of path following errors. Comparison 2, 3, 4, and 5 from the VE and RE using eye tracker.

| **Comparison** | | | | **Mean**  **[m]** | **Variance**  **[m^2^]** | **t-test**  **Type** | **t** | **Critical t** | **df** | **p-Value** |
| --- | --- | --- | --- | --- | --- | --- | --- | --- | --- | --- |
| **#** | | **Between** | |  |  |  |  |  |  |  |
|  | 2 |  | VET T6 | 0.339 | 0.001 | Paired  one-tailed | 2.205 | 2.353 | 3 | 0.057 |
|  |  |  | VET T1 | 0.279 | 0.005 |  |  |  |  |  |
|  | 3 |  | RET VT | 0.380 | 0.012 | Paired  two-tailed | 0.887 | 3.182 | 3 | 0.440 |
|  |  |  | VET T6 | 0.339 | 0.001 |  |  |  |  |  |
|  | 4 |  | RET VT | 0.350 | 0.013 | Paired  one-tailed | 0.989 | 2.132 | 4 | 0.189 |
|  |  |  | VET T1 | 0.296 | 0.005 |  |  |  |  |  |
|  | 5 |  | RET VT | 0.350 | 0.013 | Unpaired  one-tailed  equal  variances | 1.517 | 1.860 | 8 | 0.084 |
|  |  |  | RET T1 | 0.264 | 0.003 |  |  |  |  |  |
|  | 2 |  | RET T6 | 0.444 | 0.001 | Paired  one-tailed | 7.164 | 2.353 | 3 | 0.003 |
|  |  |  | RET T1 | 0.254 | 0.004 |  |  |  |  |  |
|  | 3 |  | RET T6 | 0.444 | 0.001 | Paired  two-tailed | 1.624 | 3.182 | 3 | 0.203 |
|  |  |  | VET RT | 0.358 | 0.007 |  |  |  |  |  |
|  | 4 |  | VET RT | 0.352 | 0.005 | Paired  one-tailed | 2.385 | 2.132 | 4 | 0.038 |
|  |  |  | RET T1 | 0.264 | 0.003 |  |  |  |  |  |
|  | 5 |  | VET RT | 0.352 | 0.005 | Unpaired  one-tailed  equal  variances | 1.207 | 1.860 | 8 | 0.131 |
|  |  |  | VET T1 | 0.296 | 0.005 |  |  |  |  |  |

E. Commands in Virtual and Real Training Using a Joystick.

**Table S17.** Total number of commands made in virtual training using a joystick.

| **Trial** | | **Participants from the VJ Group** | | | | | **Mean** | **SD** |
| --- | --- | --- | --- | --- | --- | --- | --- | --- |
|  |  | **1** | **2** | **3** | **4** | **5** |  |  |
|  | VJ T1 | 36 | 37 | 42 | 35 | 55 | **41.00** | 7.40 |
|  | VJ T2 | 31 | 37 | 28 | 32 | 46 | **34.80** | 6.31 |
|  | VJ T3 | 33 | 43 | 34 | 34 | 49 | **38.60** | 6.34 |
|  | VJ T4 | 46 | 45 | 18 | 41 | 46 | **39.20** | 10.76 |
|  | VJ T5 | 37 | 26 | 29 | 35 | 35 | **32.40** | 4.18 |
|  | VJ T6 | 35 | 37 | 35 | 38 | 42 | **37.40** | 2.58 |
|  | RJ VT | 26 | 33 | 49 | 41 | 42 | **38.20** | 7.93 |

**Table S18.** Total number of commands made in real training using a joystick.

| **Trial** | | **Participants from the RJ Group** | | | | | **Mean** | **SD** |
| --- | --- | --- | --- | --- | --- | --- | --- | --- |
|  |  | **6** | **7** | **8** | **9** | **10** |  |  |
|  | RJ T1 | 15 | 27 | 31 | 46 | 34 | **30.60** | 10.05 |
|  | RJ T2 | 11 | 33 | 31 | 28 | 22 | **25.00** | 7.92 |
|  | RJ T3 | 11 | 13 | 25 | 38 | 20 | **21.40** | 9.69 |
|  | RJ T4 | 13 | 11 | 31 | 31 | 20 | **21.20** | 8.54 |
|  | RJ T5 | 11 | - | 24 | 25 | 13 | **18.25** | 6.30 |
|  | RJ T6 | 13 | - | 23 | 23 | 11 | **17.50** | 5.55 |
|  | VJ RT | 31 | 32 | 73 | 35 | 47 | **43.60** | 15.77 |

**Table S19.** Statistical test for the number of commands made in virtual and real training with a joystick (Comparison 1).

| **Trial** | **1** | **2** | **3** | **4** | **5** | **6** |
| --- | --- | --- | --- | --- | --- | --- |
| SW | 0.838 | 0.886 | 0.892 | 0.862 | 0.922 | 0.945 |
| Critical Wα | 0.806 | 0.806 | 0.806 | 0.806 | 0.806 | 0.806 |
| **p-value** | **0.159** | **0.337** | **0.369** | **0.236** | **0.548** | **0.686** |
| F | 1.844 | 1.579 | 2.332 | 1.587 | 2.427 | 4.940 |
| N df | 4 | 4 | 4 | 4 | 3 | 3 |
| D df | 4 | 4 | 4 | 4 | 4 | 4 |
| **p-value** | **0.284** | **0.334** | **0.216** | **0.333** | **0.206** | **0.078** |
| t | 1.666 | 1.935 | 2.971 | 2.621 | 3.559 | 6.280 |
| df | 8 | 8 | 8 | 8 | 7 | 7 |
| **p-value** | **0.1342** | **0.0890** | **0.0178** | **0.0306** | **0.0092** | **0.0004** |

**Table S20.** T-test of total number of commands made. Comparison 2, 3, 4, and 5 from the VE and RE using a joystick.

| **Comparison** | | | | **Mean**  **[commands]** | **Variance**  **[commands^2^]** | **t-test**  **Type** | **t** | **Critical**  **t** | **df** | **p-Value** |
| --- | --- | --- | --- | --- | --- | --- | --- | --- | --- | --- |
| **#** | **Between** | | |  |  |  |  |  |  |  |
|  | 2 |  | VJ T1 | 41.0 | 68.5 | Paired  one-tailed | 1.260 | 2.13 | 4 | 0.138 |
|  |  |  | VJ T6 | 37.4 | 8.3 |  |  |  |  |  |
|  | 3 |  | RJ VT | 38.2 | 78.7 | Paired  two-tailed | 0.207 | 2.78 | 4 | 0.846 |
|  |  |  | VJ T6 | 37.4 | 8.3 |  |  |  |  |  |
|  | 4 |  | VJ T1 | 41.0 | 68.5 | Paired  one-tailed | 0.688 | 2.13 | 4 | 0.265 |
|  |  |  | RJ VT | 38.2 | 78.7 |  |  |  |  |  |
|  | 5 |  | RJ VT | 38.2 | 78.7 | Unpaired  one-tailed  unequal  variances | 1.187 | 1.86 | 8 | 0.135 |
|  |  |  | RJ T1 | 30.6 | 126.3 |  |  |  |  |  |
|  | 2 |  | RJ T1 | 34.5 | 67.0 | Paired  one-tailed | 4.627 | 2.35 | 3 | 0.010 |
|  |  |  | RJ T6 | 17.5 | 41.0 |  |  |  |  |  |
|  | 3 |  | VJ RT | 46.5 | 358.3 | Paired  two-tailed | 3.349 | 3.18 | 3 | 0.044 |
|  |  |  | RJ T6 | 17.5 | 41.0 |  |  |  |  |  |
|  | 4 |  | VJ RT | 43.6 | 310.8 | Paired  one-tailed | 1.506 | 2.13 | 4 | 0.103 |
|  |  |  | RJ T1 | 30.6 | 126.3 |  |  |  |  |  |
|  | 5 |  | VJ RT | 43.6 | 310.8 | Unpaired  one-tailed  equal  variances | 0.299 | 1.86 | 8 | 0.386 |
|  |  |  | VJ T1 | 41.0 | 68.5 |  |  |  |  |  |

F. Commands in Virtual and Real Training Using Eye Tracker.

**Table S21.** Total number of commands made in virtual training using eye tracker.

| **Trial** | | **VET Participant** | | | | | **Mean** | **SD** |
| --- | --- | --- | --- | --- | --- | --- | --- | --- |
|  |  | **11** | **12** | **13** | **14** | **15** |  |  |
|  | VET T1 | 57 | 45 | 69 | 50 | 48 | **53.80** | 8.57 |
|  | VET T2 | 58 | 50 | 54 | 51 | 43 | **51.20** | 4.96 |
|  | VET T3 | 50 | 45 | 69 | 43 | 40 | **49.40** | 10.33 |
|  | VET T4 | 57 | 44 | 60 | 41 | 38 | **48.00** | 8.83 |
|  | VET T5 | 45 | 44 | 78 | 42 | - | **52.25** | 14.91 |
|  | VET T6 | 44 | 44 | 57 | 44 | - | **47.25** | 5.63 |
|  | RET VT | 64 | 53 | 66 | 48 | 56 | **57.40** | 6.74 |

**Table S22.** Total number of commands made in real training using eye tracker.

| **Trial** | | **RET Participant** | | | | | **Mean** | **SD** |
| --- | --- | --- | --- | --- | --- | --- | --- | --- |
|  |  | **16** | **17** | **18** | **19** | **20** |  |  |
|  | RET T1 | 89 | 53 | 52 | 52 | 81 | **65.40** | 16.21 |
|  | RET T2 | 78 | 48 | 60 | 57 | 55 | **59.60** | 10.01 |
|  | RET T3 | 73 | 48 | 43 | 54 | 66 | **56.80** | 11.16 |
|  | RET T4 | 62 | 51 | 60 | 60 | 60 | **58.60** | 3.88 |
|  | RET T5 | 72 | 54 | 52 | 54 | 57 | **57.80** | 7.28 |
|  | RET T6 | 56 | 57 | - | 63 | 71 | **61.75** | 5.97 |
|  | VET RT | 41 | 50 | 37 | 45 | 42 | **43.60** | 15.77 |

**Table S23.** Statistical test for the number of commands made in virtual and real training with eye tracker (Comparison 1).

| **Trial** | **1** | **2** | **3** | **4** | **5** | **6** |
| --- | --- | --- | --- | --- | --- | --- |
| SW | 0.911 | 0.967 | 0.893 | 0.907 | 0.891 | 0.855 |
| Critical Wα | 0.806 | 0.806 | 0.806 | 0.806 | 0.806 | 0.806 |
| **p-value** | **0.476** | **0.852** | **0.373** | **0.451** | **0.387** | **0.253** |
| F | 3.580 | 4.081 | 1.168 | 5.186 | 4.475 | 1.126 |
| Num df | 4 | 4 | 4 | 4 | 3 | 3 |
| Den df | 4 | 4 | 4 | 4 | 4 | 3 |
| **p-value** | **0.122** | **0.101** | **0.442** | **0.070** | **0.091** | **0.462** |
| t | 1.266 | 1.504 | 0.973 | 2.198 | 0.644 | 3.060 |
| df | 8 | 8 | 8 | 8 | 7 | 6 |
| **p-value** | **0.241** | **0.171** | **0.359** | **0.059** | **0.540** | **0.022** |

**Table S24.** T-test of total number of commands made. Comparison 2, 3, 4, and 5 from the VE and RE using eye tracker.

| **Comparison** | | | | **Mean**  **[commands]** | **Variance**  **[commands^2^]** | **t-test**  **Type** | **t** | **Critical**  **t** | **df** | **p-Value** |
| --- | --- | --- | --- | --- | --- | --- | --- | --- | --- | --- |
| # | | **Between** | |  |  |  |  |  |  |  |
|  | 2 |  | VET T1 | 55.3 | 108.3 | Paired  one-tailed | 2.858 | 2.35 | 3 | 0.032 |
|  |  |  | VET T6 | 47.3 | 42.3 |  |  |  |  |  |
|  | 3 |  | RET VT | 57.8 | 74.9 | Paired  two-tailed | 3.108 | 3.18 | 3 | 0.053 |
|  |  |  | VET T6 | 47.3 | 42.3 |  |  |  |  |  |
|  | 4 |  | VET T1 | 53.8 | 91.7 | Paired  one-tailed | -1.439 | 2.13 | 4 | 0.112 |
|  |  |  | RET VT | 57.4 | 56.8 |  |  |  |  |  |
|  | 5 |  | RET T1 | 65.4 | 328.3 | Unpaired one-tailed unequal variances | 0.912 | 1.86 | 8 | 0.194 |
|  |  |  | RET VT | 57.4 | 56.8 |  |  |  |  |  |
|  | 2 |  | RET T6 | 61.8 | 47.6 | Paired  one-tailed | 0.509 | 2.35 | 3 | 0.323 |
|  |  |  | RET T1 | 59.5 | 205.7 |  |  |  |  |  |
|  | 3 |  | RET T6 | 61.8 | 47.6 | Paired  two-tailed | 3.789 | 3.18 | 3 | 0.032 |
|  |  |  | VET RT | 44.5 | 16.3 |  |  |  |  |  |
|  | 4 |  | RET T1 | 65.4 | 328.3 | Paired  one-tailed | 2.505 | 2.13 | 4 | 0.033 |
|  |  |  | VET RT | 43.0 | 23.5 |  |  |  |  |  |
|  | 5 |  | VET T1 | 53.8 | 91.7 | Unpaired one-tailed equal variances | 2.250 | 1.86 | 8 | 0.027 |
|  |  |  | VET RT | 43.0 | 23.5 |  |  |  |  |  |

G. Sense of Presence Questionnaire Results From All Participants.

**Table S25.** Question for each IPQ item.

| **Item Name** | | **Questions** |
| --- | --- | --- |
|  | G1 | In the computer-generated world, I had a sense of "being there". |
|  | SP1 | Somehow, I felt that the virtual world surrounded me. |
|  | SP2***** | I felt like I was just perceiving pictures. |
|  | SP3 | I did not feel present in the virtual space. |
|  | SP4 | I had a sense of acting in the virtual space, rather than operating something from outside. |
|  | SP5 | I felt present in the virtual space. |
|  | INV1 | How aware were you of the real world surrounding while navigating in the virtual world? (i.e. sounds, room temperature, other people, etc.)? |
|  | INV2 | I was not aware of my real environment. |
|  | INV3* | I still paid attention to the real environment. |
|  | INV4 | I was completely captivated by the virtual world. |
|  | REAL1* | How real did the virtual world seem to you? |
|  | REAL2 | How much did your experience in the virtual environment seem consistent with your real-world experience? |
|  | REAL3 | How real did the virtual world seem to you? |
|  | REAL4 | The virtual world seemed more realistic than the real world. |

**Table S26.** Results for each IPQ item after driving the virtual EPW using an HMD and a joystick.

| **IPQ Item** | | | **VJ Participants** | | | | | **RJ Participants** | | | | | **Mean** | **SD** |
| --- | --- | --- | --- | --- | --- | --- | --- | --- | --- | --- | --- | --- | --- | --- |
| **#** | **Name** | | **1** | **2** | **3** | **4** | **5** | **6** | **7** | **8** | **9** | **10** |  |  |
| 1 |  | G1 | 5 | 6 | 5 | 4 | 6 | 5 | 5 | 6 | 6 | 6 | 5.40 | 0.66 |
| 2 |  | SP1 | 6 | 6 | 4 | 6 | 6 | 6 | 5 | 6 | 5 | 5 | 5.50 | 0.67 |
| 3 |  | SP2***** | 6 | 6 | 5 | 6 | 5 | 5 | 4 | 6 | 4 | 5 | 5.20 | 0.75 |
| 4 |  | SP3 | 5 | 3 | 3 | 6 | 6 | 5 | 5 | 6 | 5 | 6 | 5.00 | 1.10 |
| 5 |  | SP4 | 5 | 6 | 5 | 6 | 6 | 6 | 4 | 6 | 6 | 6 | 5.60 | 0.66 |
| 6 |  | SP5 | 5 | 6 | 5 | 6 | 6 | 5 | 4 | 6 | 5 | 6 | 5.40 | 0.66 |
| 7 |  | INV1 | 5 | 6 | 6 | 5 | 2 | 3 | 4 | 6 | 4 | 6 | 4.70 | 1.35 |
| 8 |  | INV2 | 5 | 6 | 6 | 5 | 4 | 3 | 4 | 6 | 4 | 6 | 4.90 | 1.04 |
| 9 |  | INV3* | 5 | 6 | 5 | 1 | 2 | 3 | 2 | 2 | 3 | 6 | 3.50 | 1.75 |
| 10 |  | INV4 | 5 | 6 | 4 | 6 | 5 | 3 | 5 | 6 | 4 | 6 | 5.00 | 1.00 |
| 11 |  | REAL1* | 3 | 3 | 4 | 5 | 5 | 5 | 5 | 3 | 5 | 6 | 4.40 | 1.02 |
| 12 |  | REAL2 | 5 | 2 | 2 | 6 | 6 | 5 | 5 | 3 | 5 | 6 | 4.50 | 1.50 |
| 13 |  | REAL3 | 3 | 3 | 3 | 6 | 5 | 5 | 5 | 4 | 5 | 6 | 4.50 | 1.12 |
| 14 |  | REAL4 | 0 | 1 | 0 | 3 | 3 | 2 | 3 | 0 | 2 | 2 | 1.60 | 1.20 |

**Table S27.** Mean and standard deviation of each IPQ factor after driving the virtual EPW using an HMD and a joystick.

| **IPQ Item Name** | | **Mean** | **SD Pooled** |
| --- | --- | --- | --- |
|  | G1 | 5.40 | 0.66 |
|  | SP | 5.34 | 0.60 |
|  | INV | 4.53 | 1.02 |
|  | REAL | 3.75 | 1.35 |

**Table S28.** Results for each IPQ item after driving the virtual EPW using a projector and eye tracker.

| **IPQ Item** | | | **VET Participants** | | | | | **RET Participants** | | | | | **Mean** | **SD** |
| --- | --- | --- | --- | --- | --- | --- | --- | --- | --- | --- | --- | --- | --- | --- |
| **#** | **Name** | | **1** | **2** | **3** | **4** | **5** | **6** | **7** | **8** | **9** | **10** |  |  |
| 1 |  | G1 | 3 | 5 | 2 | 5 | 5 | 3 | 4 | 3 | 5 | 5 | 4.00 | 1.10 |
| 2 |  | SP1 | 4 | 5 | 1 | 3 | 5 | 3 | 3 | 5 | 5 | 5 | 3.90 | 1.30 |
| 3 |  | SP2***** | 1 | 5 | 3 | 2 | 2 | 3 | 5 | 6 | 6 | 5 | 3.80 | 1.72 |
| 4 |  | SP3 | 4 | 5 | 2 | 1 | 4 | 4 | 5 | 6 | 5 | 5 | 4.10 | 1.45 |
| 5 |  | SP4 | 4 | 5 | 4 | 2 | 4 | 4 | 5 | 3 | 5 | 5 | 4.10 | 0.94 |
| 6 |  | SP5 | 3 | 5 | 3 | 3 | 5 | 5 | 5 | 6 | 5 | 5 | 4.50 | 1.02 |
| 7 |  | INV1 | 4 | 3 | 1 | 6 | 3 | 2 | 2 | 6 | 4 | 5 | 3.60 | 1.62 |
| 8 |  | INV2 | 5 | 3 | 1 | 5 | 2 | 1 | 2 | 6 | 4 | 5 | 3.40 | 1.74 |
| 9 |  | INV3* | 4 | 1 | 2 | 4 | 1 | 1 | 4 | 6 | 5 | 5 | 3.30 | 1.79 |
| 10 |  | INV4 | 3 | 5 | 0 | 5 | 4 | 1 | 4 | 6 | 5 | 5 | 3.80 | 1.83 |
| 11 |  | REAL1* | 4 | 5 | 3 | 2 | 5 | 2 | 5 | 4 | 5 | 5 | 4.00 | 1.18 |
| 12 |  | REAL2 | 5 | 4 | 4 | 2 | 5 | 4 | 4 | 5 | 6 | 5 | 4.40 | 1.02 |
| 13 |  | REAL3 | 4 | 5 | 4 | 2 | 5 | 4 | 3 | 5 | 5 | 5 | 4.20 | 0.98 |
| 14 |  | REAL4 | 3 | 3 | 0 | 0 | 2 | 0 | 0 | 1 | 1 | 1 | 1.10 | 1.14 |

**Table S29.** Mean and standard deviation of each IPQ factor after driving the virtual EPW using a projector and eye tracker.

| **IPQ Item Name** | | **Mean** | **SD Pooled** |
| --- | --- | --- | --- |
|  | G1 | 4.00 | 1.10 |
|  | SP | 4.08 | 0.83 |
|  | INV | 3.53 | 0.79 |
|  | REAL | 3.43 | 1.48 |

H. User Experience Questionnaire Results From All Participants.

**Table S30.** Results of participants’ agreement-disagreement level for each user experience question for the groups that used a joystick in the VE and RE.

| **Participant** | | **Scores for the Questions** | | | | | | | | | |
| --- | --- | --- | --- | --- | --- | --- | --- | --- | --- | --- | --- |
| **Group** | **#** | **1** | **2** | **3** | **4** | **5** | **6** | **7** | **8** | **9** | **10** |
| **VJ** | 1 | 4 | 5 | 4 | 1 | 1 | 5 | - | 3 | - | 2 |
|  | 2 | 4 | 5 | 5 | 1 | 1 | 3 | - | 5 | - | 4 |
|  | 3 | 3 | 4 | 4 | 2 | 1 | 5 | - | 3 | - | 5 |
|  | 4 | 4 | 4 | 5 | 1 | 1 | 5 | - | 1 | - | 5 |
|  | 5 | 4 | 4 | 5 | 1 | 1 | 4 | - | 5 | - | 5 |
| **RJ** | 6 | 4 | 4 | 4 | 1 | 1 | - | 5 | 4 | - | 3 |
|  | 7 | 4 | 4 | 4 | 3 | 3 | - | 4 | 2 | - | 2 |
|  | 8 | 5 | 3 | 5 | 1 | 1 | - | 5 | 3 | - | 1 |
|  | 9 | 4 | 5 | 5 | 1 | 1 | - | 5 | 3 | - | 2 |
|  | 10 | 5 | 1 | 4 | 4 | 2 | - | 2 | 2 | - | 1 |

**Table S31.** Results of participants’ agreement-disagreement level for each user experience question for the groups that used eye tracker in the VE and RE.

| **Participant** | | **Scores for the Questions** | | | | | | | | | |
| --- | --- | --- | --- | --- | --- | --- | --- | --- | --- | --- | --- |
| **Group** | **#** | **1** | **2** | **3** | **4** | **5** | **6** | **7** | **8** | **9** | **10** |
| **VET** | 11 | 4 | 4 | 1 | 1 | 4 | - | 5 | - | 2 | 5 |
|  | 12 | 4 | 4 | 3 | 1 | 3 | - | 5 | - | 2 | 5 |
|  | 13 | 4 | 5 | 4 | 1 | 3 | - | 5 | - | 4 | 5 |
|  | 14 | 5 | 4 | 4 | 1 | 2 | - | 5 | - | 5 | 5 |
|  | 15 | 5 | 5 | 4 | 1 | 3 | - | 5 | - | 4 | 5 |
| **RET** | 16 | 5 | 5 | 4 | 1 | 1 | 5 | - | - | 4 | 5 |
|  | 17 | 4 | 4 | 4 | 2 | 3 | 4 | - | - | 4 | 1 |
|  | 18 | 1 | 4 | 3 | 2 | 4 | 5 | - | - | 5 | 5 |
|  | 19 | - | 2 | 2 | 4 | 4 | 5 | - | - | 1 | 5 |
|  | 20 | 4 | 5 | 4 | 2 | 3 | 5 | - | - | 2 | 5 |
